# A feedback loop between dipeptide-repeat protein, TDP-43 and karyopherin-α mediates *C9orf72*-related neurodegeneration

**DOI:** 10.1093/brain/awy241

**Published:** 2018-09-25

**Authors:** Daniel A Solomon, Alan Stepto, Wing Hei Au, Yoshitsugu Adachi, Danielle C Diaper, Rachel Hall, Anjeet Rekhi, Adel Boudi, Paraskevi Tziortzouda, Youn-Bok Lee, Bradley Smith, Jessika C Bridi, Greta Spinelli, Jonah Dearlove, Dickon M Humphrey, Jean-Marc Gallo, Claire Troakes, Manolis Fanto, Matthias Soller, Boris Rogelj, Richard B Parsons, Christopher E Shaw, Tibor Hortobágyi, Frank Hirth

**Affiliations:** 1 King’s College London, Department of Basic and Clinical Neuroscience, Maurice Wohl Clinical Neuroscience Institute, Institute of Psychiatry, Psychology and Neuroscience, London, UK; 2 School of Biosciences, College of Life and Environmental Sciences, University of Birmingham, Edgbaston, Birmingham, UK; 3 Jozef Stefan Institute, Department of Biotechnology and Biomedical Research Institute BRIS and University of Ljubljana, Faculty of Chemistry and Chemical Technology, Ljubljana, Slovenia; 4 King’s College London, School of Cancer Studies and Pharmaceutical Sciences, London, UK; 5 MTA-DE Cerebrovascular and Neurodegenerative Research Group, Departments of Neurology and Neuropathology, University of Debrecen, Debrecen, Hungary; 6 King’s College London, Department of Old Age Psychiatry, Institute of Psychiatry, Psychology and Neuroscience, London, UK

**Keywords:** amyotrophic lateral sclerosis, frontotemporal dementia, TDP-43, C9ORF72, karyopherin

## Abstract

Accumulation and aggregation of TDP-43 is a major pathological hallmark of amyotrophic lateral sclerosis and frontotemporal dementia. TDP-43 inclusions also characterize patients with GGGGCC (G4C2) hexanucleotide repeat expansion in *C9orf72* that causes the most common genetic form of amyotrophic lateral sclerosis and frontotemporal dementia (C9ALS/FTD). Functional studies in cell and animal models have identified pathogenic mechanisms including repeat-induced RNA toxicity and accumulation of G4C2-derived dipeptide-repeat proteins. The role of TDP-43 dysfunction in C9ALS/FTD, however, remains elusive. We found G4C2-derived dipeptide-repeat protein but not G4C2-RNA accumulation caused TDP-43 proteinopathy that triggered onset and progression of disease in *Drosophila* models of C9ALS/FTD. Timing and extent of TDP-43 dysfunction was dependent on levels and identity of dipeptide-repeat proteins produced, with poly-GR causing early and poly-GA/poly-GP causing late onset of disease. Accumulating cytosolic, but not insoluble aggregated TDP-43 caused karyopherin-α2/4 (KPNA2/4) pathology, increased levels of dipeptide-repeat proteins and enhanced G4C2-related toxicity. Comparable KPNA4 pathology was observed in both sporadic frontotemporal dementia and C9ALS/FTD patient brains characterized by its nuclear depletion and cytosolic accumulation, irrespective of TDP-43 or dipeptide-repeat protein aggregates. These findings identify a vicious feedback cycle for dipeptide-repeat protein-mediated TDP-43 and subsequent KPNA pathology, which becomes self-sufficient of the initiating trigger and causes C9-related neurodegeneration.

## Teaser

TDP-43 accumulation is a major pathological hallmark of amyotrophic lateral sclerosis and frontotemporal dementia, including the most common genetic cause, G4C2 hexanucleotide repeat expansion in C9ORF72 (C9ALS/FTD). Solomon *et al*. report that G4C2-derived dipeptide repeat protein but not G4C2-RNA accumulation causes TDP-43 proteinopathy that triggers onset and progression of disease.

## Introduction

Amyotrophic lateral sclerosis (ALS) and frontotemporal dementia (FTD) are devastating neurodegenerative diseases for which no cure is available (Van Langenhove *et al.*, 2012; Ling *et al.*, 2013). Genetic evidence suggests that both diseases form part of a clinical spectrum, most prominently underpinned by a large GGGGCC (G4C2) expansion in intron 1 of chromosome 9 open reading frame 72 (*C9orf72*) (DeJesus-Hernandez *et al.*, 2011; Renton *et al.*, 2011) that is the most common cause of ALS and FTD (C9ALS/FTD) (Stepto *et al.*, 2014; Rohrer *et al.*, 2015). In addition to genetic evidence, proteinaceous inclusions of TAR DNA-binding protein 43 (TDP-43, encoded by *TARDBP*) are a histopathological hallmark of 97% of ALS and 45% of FTD cases (Ling *et al.*, 2013).

TDP-43 is an RNA binding protein with two RNA recognition motifs (RRMs), a nuclear localization (NLS) and nuclear export signal, and a C-terminal low complexity domain with prion-like properties that harbours most of the mutations associated with familial forms of ALS and FTD (Lee *et al.*, 2011). TDP-43 shuttles between the nucleus and cytoplasm where it functions in mRNA stability, translation and transport (Lee *et al.*, 2011; Ederle and Dormann, 2017). Imbalance of this process leads to cytoplasmic accumulation and in turn nuclear depletion of TDP-43 and assembly into aggregates of phosphorylated and ubiquitinated C-terminal fragments that characterize the progressive and end stages of disease (Arai *et al.*, 2006; Neumann *et al.*, 2006).

TDP-43 proteinopathy is associated with the majority of C9ALS/FTD cases that are also characterized by RNA foci of G4C2 hexanucleotide repeats and sequestered RNA binding proteins (Haeusler *et al.*, 2016), and inclusions of dipeptide-repeat proteins (DPRs) that accumulate through repeat-associated non-ATG (RAN) translation (Ash *et al.*, 2013; Mori *et al.*, 2014). RAN translation from both sense and anti-sense G4C2 RNA can lead to poly-glycine–alanine (GA), poly-proline–alanine (PA), poly-glycine–proline (GP), poly-glycine–arginine (GR) and poly-proline–arginine (PR) proteins (Mori *et al.*, 2014). Functional studies in cell and animal models have identified pathogenic gain-of-function mechanisms including repeat-induced RNA toxicity and accumulation of DPRs (Mizielinska *et al.*, 2014; Wen *et al.*, 2014; Chew *et al.*, 2015), with recent evidence suggesting that both cause defective nucleocytoplasmic transport (NCT) and nuclear pore complex (NPC) deficits that eventually lead to age-related neurodegeneration (Freibaum *et al.*, 2015; Jovicic *et al.*, 2015; Zhang *et al.*, 2015; Boeynaems *et al.*, 2016, 2017; Lee *et al.*, 2016). Importantly, comparable NCT and NPC deficits were also reported most recently in models of TDP-43 aggregation (Chou *et al.*, 2018).

These findings suggest that G4C2/DPR-mediated NCT and/or NPC defects trigger disease formation but raise the question whether TDP-43 aggregation is a cause or consequence of it. Moreover, unlike TDP-43 pathology, neither RNA foci nor DPR distribution spatially correlate with clinical phenotype and neurodegeneration, except for poly-GR (Saberi *et al.*, 2018), which together with poly-PR has been shown to be the most toxic DPR species (Mizielinska *et al.*, 2014). Several murine models revealed RNA foci and DPRs develop early but are not sufficient to drive neurodegeneration in the absence of TDP-43 pathology (O’Rourke *et al.*, 2015; Peters *et al.*, 2015), whereas neurodegeneration was observed in conjunction with TDP-43 pathology (Chew *et al.*, 2015; Liu *et al.*, 2016). Interestingly, in one of these models TDP-43 aggregation occurred after the formation of both DPRs and RNA foci (Liu *et al.*, 2016). Furthermore, post-mortem analyses of C9ALS/FTD patients who died early (but of other causes), revealed only abundant DPR pathology, further indicating DPR pathology precedes that of TDP-43 (Proudfoot *et al.*, 2014; Baborie *et al.*, 2015; Vatsavayai *et al.*, 2016). Collectively, these data indicate that G4C2 RNA and/or DPRs act as initiating stressors causing the cytoplasmic mislocalization, aggregation and subsequent dysfunction of TDP-43 that mediates C9-related neurodegeneration, likely via NCT and/or NPC deficits.

Here we identify a vicious feedback loop that mediates C9ORF72-related neurodegeneration. Using novel *in vivo Drosophila* models of C9ALS/FTD and TDP-43 pathology, we first show that accumulation of G4C2-derived DPRs causes cytoplasmic mislocalization and accumulation of TDP-43. This in turn leads to nuclear depletion of karyopherin-α (KPNAs) resulting in a vicious cycle of increasing TDP-43 and KPNA mislocalization and dysfunction, which is crucially also observed in C9ALS/FTD and sporadic FTD post-mortem brain tissue.

## Materials and methods

For detailed materials and methods, please see the online Supplementary material.

### Fly stocks and husbandry

Fly stocks were maintained at 25°C on standard cornmeal food, unless for ageing experiments where flies were maintained on 15% sugar/yeast medium. Strains used were *Oregon R*; *w^1118^*; *Actin-Gal4*; *GMR-Gal4*; *Elav^C155^-Gal4*; *Elav^C155^*;*UAS-mCD8-GFP*; *Gal80ts*; *Fkh-Gal4*; *UAS-mCD8::GFP*; *UAS-hTDP-43*; *UAS-Q331K-TDP-43* (Elden *et al.*, 2010); *UAS- ΔRRM1-TDP-43* (Ihara *et al.*, 2013); and *gTBPH.*

### Generation of transgenic flies

The following UAS-attB constructs were generated: *UAS-DsRed2*, *UAS-DsRed2-(G4C2)8A*,*UAS-DsRed2-(G4C2)32*, *UAS-DsRed2-(G4C2)56*, *UAS-DsRed2-(G4C2)64*, *UAS-DsRed2-(G4C2)128B*, *UAS-DsRed2-(G4C2)8B*, *UAS-DsRed2-(G4C2)38*, *UAS-GA8*, *UAS-GA64*, *UAS-GR8*, *UAS-GR64*, *UAS-PR8*, *UAS-PR64*, *UAS-PA8*, *UAS-PA64.* G4C2 constructs were established that contained DsRed2 upstream of either (G4C2)8, (G4C2)38 or (G4C2)56. To generate plasmids containing longer repeat lengths, a further plasmid containing (G4C2)4 repeats within the 3′ UTR was generated. Two copies of this G4C2 plasmid were then differentially digested such that subsequent ligation created a plasmid containing a doubled G4C2 repeat sequence. This strategy was used to generate plasmids containing 8, 16, 32, 64 and 128 G4C2 repeats. The presence and size of G4C2 DNA within the fly genome was confirmed by Southern blotting, which produced expected results for all UAS lines except those containing 128 repeats, which displayed repeat instability (Supplementary Fig. 1B) and were thus excluded from further analysis. DPR-only constructs using alternative codon sequences coding for poly-GA, poly-GR, poly-PA and poly-PR were designed for eight amino acids and 64 amino acids. Plasmid DNA was synthesized by GeneArt (Life Technologies) and sent to BestGene for generation of transgenic flies. Constructs were integrated at site ZH-86FB for UAS-DsRed2-(G4C2) constructs and attP40 for alternative codon DPR only constructs. Genomic insertion was carried out by the Cambridge transgenic fly facility (Cambridge University, UK) and BestGene, USA. Construction of UAS flies carrying full-length human TDP-43 (*UAS-hTDP-43*) and of flies carrying genomic construct *ΔNLS-TBPH* was carried out as previously described (Diaper *et al.*, 2013). The NLS mutant (gΔNLS) was generated by mutation containing PCR.

### Reverse transcription PCR

RNA was extracted from homogenized fly heads. The following primers were used to assess *TBPH* mRNA levels: forward primer: 5′-ATCTTGGATGGCTCAGAACG-3′, reverse primer: 5′-GTCGGTCTTTATTCCGTTGG-3′. *RPL32* was used as a loading control with the following primers: forward primer: 5′-CGCCGCTTCAAGGGACAGTATC-3′, reverse primer: 5′-CGACAATCTCCTTGCGCTTCTT-3′.

### Southern and northern blotting

For Southern blotting, genomic DNA from 30 male flies per genotype was used. For northern blotting, RNA extraction from ∼70 L3 larvae was carried out. Primers specifically targeting a coding region of DsRed2 were used (forward: 5′-GTGATGCAGAAGAAGACCAT-3′ and reverse: 5′-CTTGGCCATGTAGATAGACT-3′).

### Generation of polyclonal and monoclonal poly-GP antibodies

To generate rabbit polyclonal anti-polyGP, a custom-made peptide sequence—GPGPGPGPGPGPGPGPGPGPGPGPGPGPGP (GPx15)—was fused to the C-terminus of maltose-binding protein. Two rabbits were immunized with the fusion protein and the resulting serum was purified with GST-fusion proteins containing (GP)_15_ at the C-terminus. Peptide generation and immunization was carried out by Eurogentec. Mouse monoclonal anti-polyGP (clone 2C20) was generated by immunization of mice with the custom-made peptide sequence, GPGPGPGPGPGPGPGPGPGP (GPx10), done by Abmart.

### Western blotting

For *Drosophila* samples, heads from mated female flies were removed by snap freezing in liquid nitrogen and were then homogenized in RIPA buffer. Total protein concentration was measured using the BCA kit (Thermo Scientific). For detection of insoluble protein, samples were lysed in RIPA buffer and then spun at 16 000*g* for 10 min, after which the supernatants were separated from the pellets and prepared for western blot. The pellets were washed three times with RIPA buffer then resuspended in urea buffer. For details see the Supplementary material. Antibodies used were: rabbit anti-DsRed2 (1:1000, Clontech); mouse monoclonal anti-GP (1:1000, this work); mouse monoclonal anti-GA (clone 5E9) (1:1000, Merck Millipore, MABN889); rabbit polyclonal anti-TBPH (1:2000) (Diaper *et al.*, 2013); mouse anti-TARDBP 2E2-D3 (1:100, Abnova); rabbit anti-beta actin polyclonal (1:1000, Abcam); mouse anti-beta tubulin monoclonal (1:600, DSHB); goat anti-KPNA4 (1:750, Novus); rabbit anti-GAPDH (1:5000; Cell Signaling Technology). The following secondary antibodies and dilutions were used: polyclonal goat anti-rabbit IgG (H&L) conjugated IRDye800 (Rockland immunochemicals) used at 1:10 000 and polyclonal goat anti-mouse IgG (H&L) conjugated Alexa Fluor® 680 (Thermo Fisher) used at 1:10 000.

### Dot blotting for dipeptide-repeat protein detection

Dot blots were used to detect DPRs using the protocol described previously (Mizielinska *et al.*, 2014). Primary antibodies were used as described (Gendron *et al.*, 2013; Mann *et al.*, 2013); for details see the Supplementary material.

### Fluorescence *in situ* hybridization

Fluorescence *in situ* hybridization (FISH) was carried out as described previously (Tran *et al.*, 2015) using an Alexa Fluor® 488-labelled (G2C4)4 RNA probe. Salivary glands were dissected and fixed in 4% PFA and probed as described for immunohistochemistry for mouse anti-phospho-RNA polymerase II 4H8 (1:500, Abcam) and secondary antibody used was Alexa Fluor® 568 (Life Technologies). For details see the Supplementary material.

### Immunohistochemistry

Larval CNS, larval salivary glands, larval and pupal eye discs, and adult brains were immunolabelled using rabbit anti-GFP 1:1000 (Invitrogen), rabbit polyclonal anti-GP (1:1000, this work), mouse monoclonal anti-GA (1:500, Merck Millipore), anti-GR rat monoclonal (1:300, Merck Millipore), mouse anti-HA clone 6E2 (1:500, Cell Signal), rabbit anti-TBPH (1:2000) (Diaper *et al.*, 2013), rabbit anti-RanGAP (1:300, kind gift from C. Staber); rabbit anti-Importin-α3 (1:300, kind gift from S. Cotterill); rabbit anti-Pendulin (1:300) and mouse anti-dRCC1 (1:10) (both kind gifts from M. Frasch); mouse monoclonal anti-MAB414 (1:500, Abcam), rabbit anti-NUP50 (1:10 000, kind gift from J. Großhans), and rabbit anti-REF2P (1:1000, kind gift from D. Contamine). Secondary antibodies conjugated to Alexa Fluor® 488, 568 and 647 (Life Technologies) were used at a final concentration of 1:150. See the online Supplementary material for details.

### Image acquisition and analysis

Images were obtained either with Motic BA400 or Leica TCS SP5 confocal microscopes. For quantification of the TBPH nuclear/cytoplasmic ratio, brains were dissected and washed in primary antibody solution. Single plane images were taken from the medulla of the adult brain always analysing the same region. Nuclear was defined by DAPI labelling, TBPH immunolabelling was distinguished by nuclear/DAPI and non-nuclear/non-DAPI. Eight brains of each genotype were used for measurements, which were carried out using ImageJ.

### Eye sectioning

Paraffin eye sections were done as previously described (Zaharieva *et al.*, 2015).

### Larval survival

Embryos were collected at 5-h intervals by replacement of fruit agar plate. L1 larvae were transferred to vials of standard cornmeal media at a density of 50 larvae per vial. Number of flies that underwent complete eclosion were counted. Percentage of flies surviving was calculated based on the original L1 population of 50.

### Larval peristalsis

L3 larvae were placed in the centre of fruit agar plates that had been left at room temperature for 30 min. L3 larvae were left to acclimatize for 1 min, after which the number of peristaltic waves that occurred in the following minute were counted.

### Startle-induced negative geotaxis

Startle-induced negative geotaxis (SING) was carried out following White *et al.* (2010).

### Video-assisted motion tracking

Activity tracking was carried out as previously described (White *et al.*, 2010). In addition, activity was defined as movement per frame above a velocity of 2 mm/s. Raster plots and activity per minute graphs were generated in MATLAB using custom script. Percentage of time active over the recording period was calculated for each fly. These data were exported to GraphPad Prism 6 for statistical analysis.

### Human post-mortem tissue analysis

Brain tissue samples were provided from the London Neurodegenerative Diseases Brain Bank (King’s College London, UK). Consent for autopsy, neuropathological assessment and research were obtained and all studies were carried out under the ethical approval of the tissue bank (08/MRE09/38+5). Block taking for histological and immunohistochemical studies and neuropathological assessment for neurodegenerative diseases was performed in accordance with standard criteria. Fifteen cases were used for the western blot experiments, five FTLD-TDP without the *C9orf72* expansion, five FTLD-TDP with confirmed *C9orf72* expansion and five control cases. Controls were defined as subjects with no clinical history and no neuropathological evidence of a neurodegenerative condition.

For western blot analysis, fresh-frozen post-mortem tissue was homogenized in 10 v/w of high salt buffer [100 mM 2-(N-morpholino) ethane sulphonic acid (MES) (pH 7.4), 0.5 mM MgCl_2_, 1 mM EGTA, 1 M NaCl, 50 mM imidazole, protease inhibitor cocktail (Roche)]. The homogenate was mixed with 2% SDS-PAGE loading buffer and boiled for 10 min. Samples were centrifuged for 20 min at 13 000 rpm and 4°C. Equal volumes of samples were loaded on 26-well NuPAGE® Novex 10% Bis–Tris pre-cast gels (Invitrogen). Western blots were performed as described (Nishimura *et al.*, 2010). For detection of insoluble protein, the same protocol was used as for fly tissue.

For immunohistochemistry and immunofluorescence of post-mortem human brain samples, the protocols were carried out as previously described (Maekawa *et al.*, 2004). Briefly, 7 µm thick sections from the frontal cortex were cut from formalin-fixed paraffin-embedded tissue blocks and sections were microwaved in citrate buffer to enhance antigen retrieval. For immunofluorescence, primary antibodies were used at the following concentrations; rabbit polyclonal anti-GP (1:1000, this study); mouse monoclonal anti-GA (1:500, Merck Millipore); anti-GR rat monoclonal (1:50, Merck Millipore); rabbit phospho-TDP-43 (1:500, pS409/410-1; 2B Scientific); mouse phospho-TDP-43 (1:500, pS409/410-1, Cosmo Bio Co., LTD); mouse anti-TARDBP 2E2-D3 (1:100, Abnova); rabbit anti-KPNA4 (1:250, Novus). Secondary antibodies conjugated to Alexa Fluor® 488, 568 and 647 (Life Technologies) were used at a final concentration of 1:250. Autofluorescence was quenched using Sudan black treatment and sections were mounted using Vectashield with DAPI. For immunohistochemistry, primary antibody rabbit anti-KPNA4 (1:500, Novus) was applied overnight at 4°C. Following washes, sections were incubated with biotinylated secondary antibody (Dako), followed by avidin:biotinylated enzyme complex (Vectastain Elite ABC kit, Vector Laboratories). Finally, sections were incubated for 5–10 min with 0.5 mg/ml 3,3′-diaminobenzidine chromogen (Sigma-Aldrich) in Tris-buffered saline (pH 7.6) containing 0.05% H_2_O_2_. Sections were counterstained with Harris’ haematoxylin. Immunostaining (morphology, location and intensity) was examined and assessed by a consultant neuropathologist (T.H.) blind to diagnosis.

### Statistical analysis

GraphPad Prism 6 was used to perform the statistical analyses indicated within the ‘Results’ section. Comparison of means was performed using either an unpaired *t*-test, one- or two-way ANOVA, or Bonferroni’s multiple comparisons test. *Post hoc* analysis was conducted using Fisher’s least significant difference (LSD) test or the Bonferroni-Holm method (Holm, 1979). For categorical data, Fisher’s exact test was preferred to Pearson’s chi-squared test due to small sample sizes in this dataset.

### Data availability

The authors confirm that the data supporting the findings of this study are available within the article and its Supplementary material.

## Results

### Novel *Drosophila* models of C9ALS/FTD produce different combinations and levels of G4C2-derived RNA and DPRs

**Figure 1:**
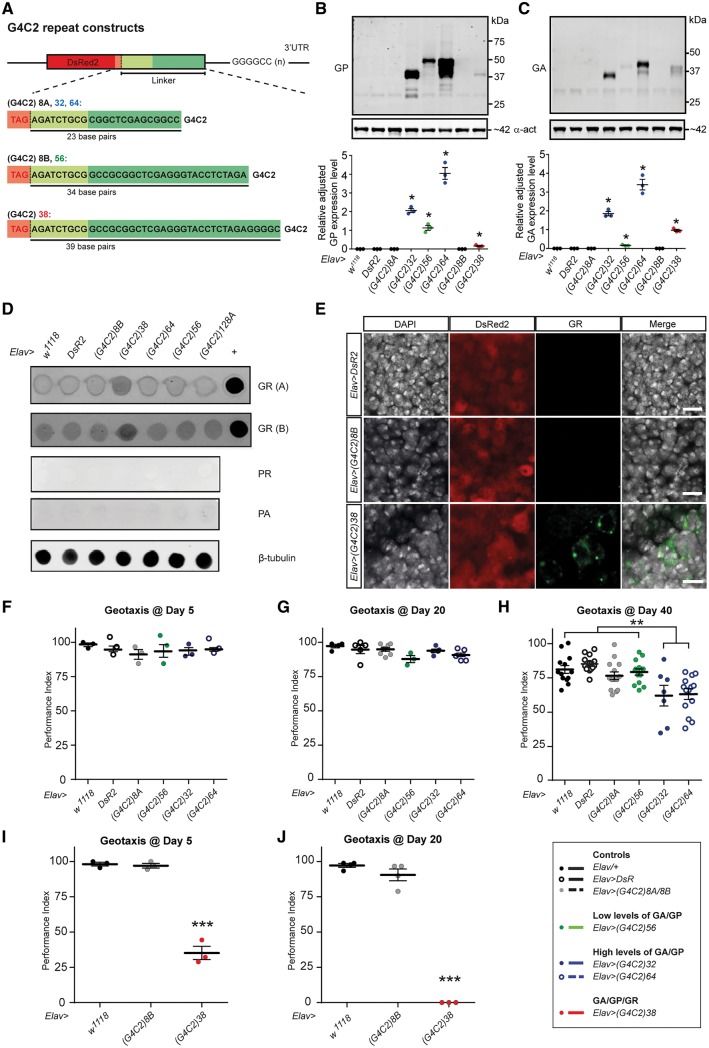
***Drosophila* C9ALS/FTD models expressing G4C2 RNA and different levels and combinations of RAN-translated DPRs.** (**A**) Schematic of constructs with different lengths of uninterrupted G4C2 repeats cloned into 3′ UTR of disease-unrelated marker gene DsRed2; variable linker sequences between DsRed2 stop codon and start of G4C2 repeats are indicated. (**B** and **C**) Quantitative western blot for poly-GP (**B**) and poly-GA (**C**) of head extracts of 5-day-old flies expressing G4C2 repeats pan-neuronally (Elav-Gal4); *P* < 0.0001; *significant difference from all other genotypes; mean with SEM shown for each genotype (*n* = 3). (**D**) Dot blot analysis of adult fly head extracts of G4C2 constructs using two published poly-GR antibodies; positive control (+) of lysate from HEK293 cells expressing poly-GR from a non-G4C2 template. No anti-sense DPRs were detected using anti-PR and anti-PA antibodies, with beta-tubulin as loading control. (**E**) Confocal images of nervous system of Elav-Gal4 mediated pan-neuronal expression of controls and G4C2 repeats. Immunohistochemistry of 5-day-old adult brains shows poly-GR detectable in flies expressing 38 G4C2 repeats but not in controls. In flies expressing 38 G4C2 repeats, poly-GR displays diffuse perinuclear localization with regions of intense staining. Scale bars = 10 µm. (**F**–**H**) Geotaxis climbing performance at Day 5 (**F**), Day 20 (**G**) and Day 40 (**H**) of flies expressing different G4C2 repeats that lead to different levels of poly-GA and poly-GP produced. Note that flies expressing 32 and 64 show motor impairment by Day 40, but not 56 G4C2 repeats nor control constructs; **P < 0.01; mean with SEM shown for each genotype (*n* = 7–14). (**I** and **J**) Geotaxis climbing performance of flies expressing 38 repeats at Day 5 (*P* < 0.001) and Day 20 (*P* < 0.001) compared to controls; mean with SEM shown for each genotype (*n* = 3–5).

To investigate G4C2/DPR-related TDP-43 pathology, novel *Drosophila* models of C9ALS/FTD, expressing different lengths of uninterrupted G4C2 repeats in the 3′ UTR of the disease-unrelated DsRed2 gene, were first created (Fig. 1A). Previous studies have shown that introduction of non-coding CAG repeats in the 3′ UTR of the DsRed2 gene, but not DsRed2 itself, is sufficient to induce age-related neurodegeneration in a sequence-specific manner (Li *et al.*, 2008). This strategy was applied to directly regulate the number of G4C2 repeats used (Supplementary Fig. 1A–C). Variable linker sequences in between the DsRed2 stop codon and start of the G4C2 repeats were introduced by chance because of the cloning strategy utilized (Fig. 1A and Supplementary Fig. 1D). Western and dot blot analyses identified sense, but not antisense DPRs, in flies expressing ≥32 repeats (Fig. 1B–D), the levels and combinations of which correlated with the corresponding linker sequence 5′ of the G4C2 repeats. High levels of poly-GP and poly-GA were observed in 32 and 64 repeat lines (Fig. 1B and C) that contain the same 5′ linker sequence; however much lower levels were seen in 38 and 56 repeat lines, both of which had their own unique 5′ linker sequence (Fig. 1A–C and Supplementary Fig. 1D). Hence DPR expression profiles were dependent on the 5′ linker sequence for G4C2 repeats of different length (Table 1) and independent of a near-cognate CUG codons 5′ of the repeats (Supplementary Fig. 1D), which has been recently related to G4C2 RAN translation (Green *et al.*, 2017; Tabet *et al.*, 2018). Of note, poly-GR was only detected in flies expressing 38 repeats (Fig. 1D and E and Supplementary Fig. 3I). The expression characteristics of these *Drosophila* models allowed us to assess the toxicity of different levels and combinations of G4C2 RNA and DPRs.

**Table 1:** Expression levels and disease onset of G4C2 repeats and derived DPRs in *Drosophila*

**G4C2 repeats**	**G4C2 level**^a^	**DPR type**	**DPR level**^b^	**Motor phenotype**
8	n/a	None	n/a	None
32	+	GA/GP	+++/+++	at Day 40
38	++	GA/GP/GR	++/+/+	at Day 5
56	+	GA/GP	+/++	not at Day 40
64	+	GA/GP	+++/+++	at Day 40
64 × 2	++	GA/GP	++++/++++	not at Day 5

^a^Based on northern blots and quantitative western blots for DsRed2 protein of DsRed2-G4C2 constructs.

^b^Based on quantitative western blots and *in situ* expression of respective DPR.

### Time of disease onset and severity depend on levels and combinations of G4C2-derived RNA/DPRs

To address the toxic potential of these different constructs, survival of larvae over-expressing G4C2 repeats under control of the pan-neuronal *Elav^C155^* driver was measured. Surprisingly, despite neuronal overexpression of potentially pathogenic G4C2 repeat lengths in all experimental genotypes, toxicity was observed in a construct-dependent manner. No significant impairment in survival was reported in flies expressing 8, 32, 56 or 64 repeats relative to flies expressing DsRed2 without G4C2 repeats (Supplementary Fig. 2). These findings suggest that G4C2 repeat RNA up to 64 repeats in length and the concomitant presence of poly-GA and poly-GP at high expression levels, are well tolerated during development. In contrast, larvae expressing 38 repeats demonstrated a significant impairment in survival relative to all other G4C2 repeat expressing larvae and negative controls. This toxicity was not due to the genomic insertion of the UAS construct as 38 repeat flies also exhibited significant survival impairment relative to the UAS control (+/DsRed2-38). To establish whether this toxicity was conserved in a different genetic background, UAS lines were backcrossed six times to *white^1118^.* Following this procedure, expression of 38 repeats remained toxic to developing larvae as evidenced by significantly impaired survival relative to all other genotypes tested (Supplementary Fig. 2).

To establish the impact of neuronal G4C2 repeat expression over time, adult locomotor performance was assessed in young (Day 5), mid-aged (Day 20) and older (Day 40) flies. Following mechanical disruption, flies were first tested for their SING response, which, after being overthrown, quantifies their ability to right themselves and climb up the test tube (White *et al.*, 2010). Analysis revealed that by Day 5, flies expressing 38 repeats displayed severely impaired climbing performance relative to all other genotypes (Fig. 1F–J). By Day 20, the poor performance in the 38 repeat flies had progressed to a complete inability to climb. By Day 40, flies expressing 38 repeats had already died, whereas a significantly impaired motor behaviour was detectable for 32 and 64 repeat flies. Comparable findings were observed with video-assisted motion tracking in an open-field assay where freely moving flies were recorded for 60 min to determine their activity bouts and movement trajectories (Supplementary Fig. 3A–E).

To rule out that these differences in toxicity were due to potential differences in Gal4/UAS expression levels (despite identical genomic insertion sites), flies were generated harbouring two copies of the 64 repeat construct that produce significantly higher levels of DsRed2 protein (Supplementary Fig. 3F). SING analysis at Day 5 of these 2 × 64 flies was compared to single copy 64 flies and the 38 repeat line. This revealed the climbing performance between 2 × 64 repeat flies and 1 × repeat 64 flies was not significantly different; however, both were significantly different to the severely impaired 38 repeat flies (Supplementary Fig. 3G). Given the construct-dependent, different G4C2 RNA and DPR expression levels, these data establish that high levels of poly-GA and poly-GP accumulation correlate with late onset, whereas poly-GR accumulation correlates with rapid onset and progression of disease (Table 1). These findings were further corroborated by enhanced age-related neurodegeneration, which by Day 35 was only observed in the 38 but not in 64 repeat or 8 repeat control flies (Supplementary Fig. 3H). Together these data establish that cellular toxicity in our C9 model is dependent on levels and identity of G4C2 RNA and/or DPRs produced rather than length of the repeat.

### DPR but not G4C2 RNA cause TDP-43 accumulation

**Figure 2:**
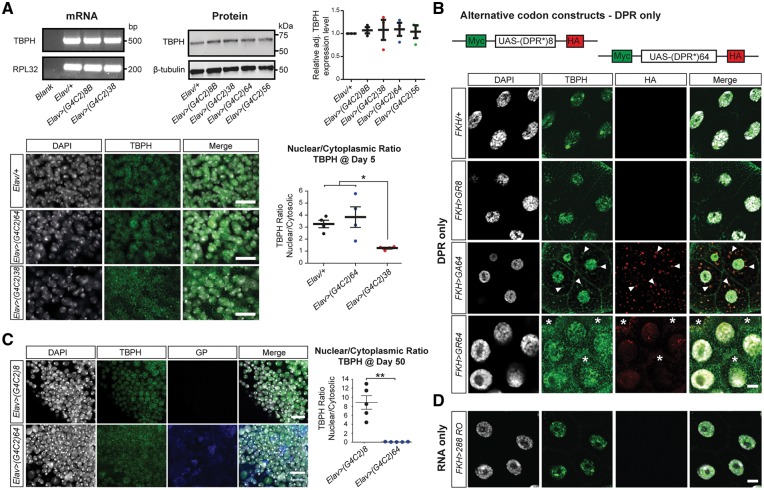
**DPR but not RNA accumulation causes cytoplasmic mislocalization of *Drosophila* TDP-43, TBPH.** (**A**) RT-PCR and quantitative western blot analyses of head extracts from 5-day-old flies expressing G4C2 repeats pan-neuronally reveals no significant effect of genotype on TBPH expression level; western blot mean with SEM shown (*n* = 3). Confocal images of Day 5 adult brains with anti-TBPH immunostaining in cytoplasm and nucleus (*n* = 4). Quantification of nuclear/cytoplasmic ratio in flies expressing 38 G4C2 repeats compared to control and flies expressing 64 G4C2 repeats; *P < 0.05, mean with SEM shown (*n* = 4). (**B**) *Top*: schematic depicting non-G4C2 alternative codon constructs designed to produce different lengths of pure DPR (8 aa and 64 aa) flanked by 5′ Myc tag and 3′ HA tag. *Bottom*: salivary glands of indicated genotypes immunolabelled with anti-TBPH and anti-HA to detect DPR distribution. Compared to controls, poly-GA64 expression causes cytoplasmic TBPH aggregates that co-localize with poly-GA inclusions (arrowheads); poly-GR64 expression leads to diffuse cytoplasmic mislocalization of TBPH (asterisks) in all cases examined (*n* = 30). Note, poly-GR64 expression also causes enlarged nuclei compared to controls. (**C**) Cytoplasmic mislocalization of TBPH is seen by Day 50 in flies expressing 64 G4C2 repeats compared to control flies expressing eight G4C2 repeats; *Right*: quantification of nuclear/cytoplasmic ratio; *P < 0.005, mean with SEM shown (*n* = 4). (**D**) Expression of G4C2 RNA only (288RO) does not alter TBPH localization compared to controls. Scale bars = 10 µm (**A** and **C**); 20 µm (**B** and **D**).

Previous studies correlated G4C2 repeats with TDP-43 mislocalization but did not distinguish between RNA and/or DPR mediated pathology and onset of disease (Freibaum *et al.*, 2015; Zhang *et al.*, 2015). We therefore investigated the expression and localization of the *Drosophila* TDP-43 homologue (Diaper *et al.*, 2013) *TBPH* in our models. No significant differences in mRNA and protein levels of TBPH were seen by Day 5, regardless of the G4C2 repeats expressed (Fig. 2A). However, by Day 5, TBPH was mislocalized to the cytosol in 38 repeat flies, but not in 64 repeat expressing flies that showed TBPH mislocalization by Day 50 (Fig. 2C), thus correlating with later onset of disease.

To distinguish between RNA and DPR mediated TBPH mislocalization, we next created transgenic flies producing different lengths of non-G4C2-derived DPR (Fig. 2B). Consistent with previous reports (Mizielinska *et al.*, 2014), expression of arginine-rich poly-GR64/PR64 but not of eight amino acid length was highly toxic (Supplementary Fig. 4). Targeted expression in development and adult-specific tissue identified 64 amino acid DPRs initially accumulated in the cytoplasm and in the case of poly-GR formed additional aggregates within 48 h in larval eye disc (Supplementary Fig. 5A) and by 18 days in adult brain neurons (Supplementary Fig. 5B). Moreover, accumulating poly-GA64 but not poly-GR64 formed inclusions that co-localized with Ref2P, the *Drosophila* homologue of p62 (Supplementary Fig. 6A). Consistent with DPR-initiated TDP-43 pathology in humans, targeted expression of non-G4C2 derived poly-GR64 and poly-GA64, caused cytoplasmic accumulation of TBPH. In all cases examined (*n* = 30) poly-GR64 expression caused an extensive diffuse cytoplasmic accumulation of TBPH (Fig. 2B, asterisks), whereas poly-GA64 expression resulted in cytoplasmic TBPH aggregates that frequently co-localized with poly-GA inclusions (Fig. 2B, arrowheads).

To rule out G4C2 RNA-mediated TBPH mislocalization, a previously characterized RNA only fly line 288RO was used, which produces high amounts of G4C2 RNA, is not subject to RAN translation and causes RNA foci formation (Mizielinska *et al.*, 2014). Of note, 288RO expression did not cause any alterations in TBPH localization, which appeared indistinguishable from controls (Fig. 2D). Moreover, Ref2P-positive inclusions were not seen in these 288RO-expressing flies (Supplementary Fig. 6A). Furthermore, with exception of a single focus identifying polymerase II activity, RNA foci were not detectable in any of our G4C2 RNA expressing flies (Supplementary Fig. 6B). Together these data demonstrate that DPRs but not G4C2 RNA foci cause cytoplasmic mislocalization of TDP-43, which correlates with onset of disease.

### Accumulating cytoplasmic TDP-43 enhances levels of RAN translated DPRs and C9-related motor impairment

Cytosolic TDP-43 functions in mRNA stability, translation and transport (Ederle and Dormann, 2017) however imbalanced cytoplasmic accumulation can induce its nuclear depletion and aggregate formation (Winton *et al.*, 2008), both of which are causally related to onset and progression of disease (Lee *et al.*, 2011; Robberecht and Philips, 2013). This dual impact of accumulation-related loss and gain of TDP-43 function raises the possibility that DPR-triggered TDP-43 dysfunction propagates C9-related toxicity.

**Figure 3:**
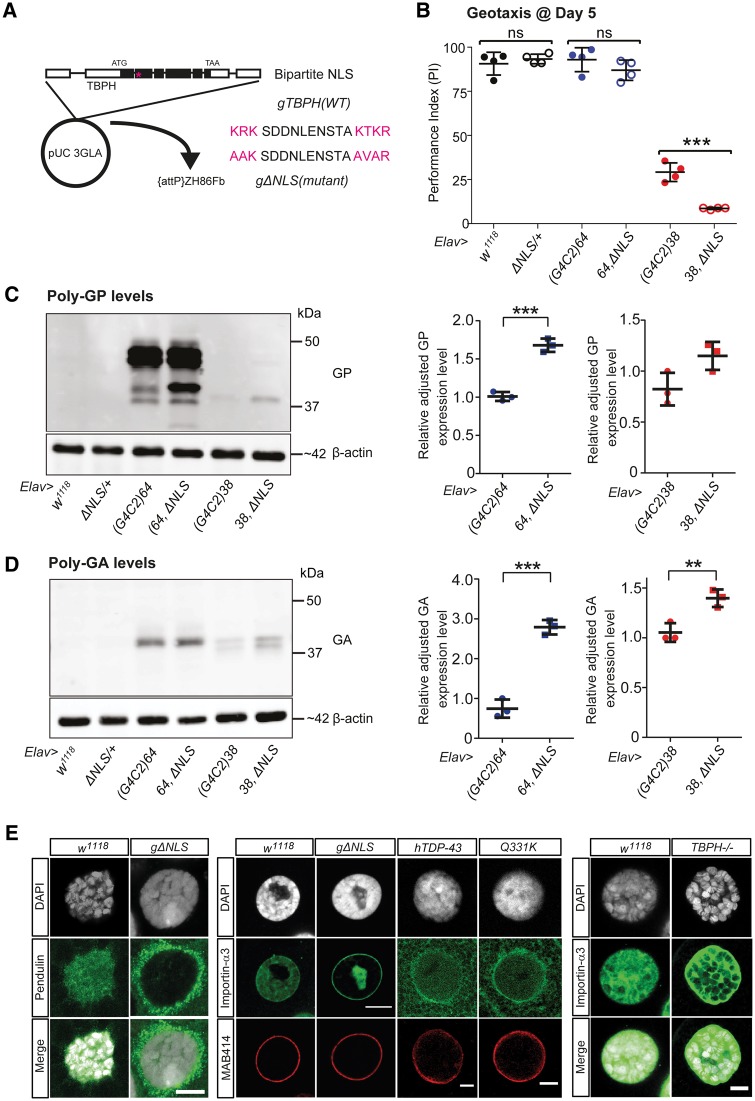
**Accumulating cytoplasmic TBPH enhances G4C2-related motor impairment and levels of DPRs produced, and causes karyopherin-α pathology.** (**A**) Schematic of genomic *TBPH* region indicating exon (black boxes) intron structure, protein coding start (ATG) and stop codon (TAA); pink asterisk indicates bipartite nuclear localization signal (NLS) for wild-type (WT) *TBPH* and mutant (ΔNLS) constructs subcloned into pUC 3GLA vector and inserted by site-specific integration at ZH86Fb on the third chromosome. (**B**) Day 5 motor performances of control flies and flies co-expressing ΔNLS-*TBPH* with 64 or 38 G4C2 repeats; ***P < 0.001, mean with SEM shown (*n* = 4). (**C** and **D**) Quantitative western blots for poly-GP (**C**) and poly-GA (**D**) using Day 5 head extracts of controls and flies co-expressing ΔNLS-*TBPH* with 64 and 38 G4C2 repeats; **P < 0.01, ***P < 0.001; mean and SEM shown (*n* = 3). (**E**) Immunostaining of importin-α3 (KPNA4) in salivary gland cells reveals loss of nuclear staining in ΔNLS-*TBPH* (gΔNLS) (*n* = 12). Also, immunostaining of pendulin (KPNA2) in salivary gland cells reveals loss of nuclear staining as well as cytoplasmic accumulation in ΔNLS-*TBPH* (gΔNLS) (*n* = 12). Comparable cytosolic mislocalization and reduced nuclear staining of importin-α3/KPNA4 can be seen in flies expressing either full-length human TDP-43 or its disease-related mutant Q331K. No nuclear depletion of importin-α3 was seen in controls or homozygous *TBPH* null mutant. MAB414 immunolabelling recognizing the conserved FG motif in Nup62, Nup153, Nup214 and Nup358 appears unaltered between controls, ΔNLS-TBPH and hTDP-43 or Q331K. Scale bars = 10 µm. ns = not significant.

To investigate this hypothesis, we generated a TBPH construct with mutated nuclear localization signal (ΔNLS-TBPH) under control of the endogenous TBPH promoter (Fig. 3A). Expression of ΔNLS-TBPH resulted in cytoplasmic accumulation and subsequent nuclear depletion of TBPH (Supplementary Fig. 7A–H), eventually causing neurodegeneration in a dose-dependent manner (Supplementary Fig. 7I). Analysis of RIPA and urea protein fractions of adult head extracts showed that TBPH toxicity was unrelated to urea-soluble aggregates (Supplementary Fig. 7J). This was independently confirmed by expressing either wild-type or aggregate forming mutant human TDP-43 missing RNA Recognition Motif 1 (ΔRRM1-TDP-43) (Ihara *et al.*, 2013), which revealed high toxicity of TDP-43 did not correlate with the formation of TDP-43-positive urea-soluble inclusions (Supplementary Fig. 7K–M). These data suggest that accumulating cytosolic but not aggregated TDP-43 causes onset of disease and C9-related toxicity.

To test this hypothesis further, we co-expressed ΔNLS-TBPH with 38 and 64 G4C2 repeats and measured the motor behaviour of these flies compared to controls. SING analysis revealed that by Day 5, no significant impairment in the motor behaviour of 64,ΔNLS-TBPH flies was detectable, but exacerbated motor impairment in 38,ΔNLS-TBPH flies at this time point when compared to 38 repeat only flies and controls (Fig. 3B). Notably, co-expression of ΔNLS-TBPH led to increased levels of DPRs in both 64 and 38 G4C2 repeat flies, as exemplified by quantitative western blotting for poly-GP (Fig. 3C) and poly-GA (Fig. 3D). Together these data demonstrate that accumulation of cytosolic TDP-43 enhances G4C2-related toxicity and increases levels of DPRs, thus identifying a vicious cycle between TDP-43 and DPR accumulation and the propagation of C9-related toxicity.

### Dipeptide-repeat protein accumulation causes KPNA but not RanGAP or nuclear pore complex pathology

Several recent studies reported NCT and NPC deficits in G4C2 RNA and DPR expression models (Freibaum *et al.*, 2015; Jovicic *et al.*, 2015; Zhang *et al.*, 2015; Boeynaems *et al.*, 2016), and most recently also in models of TDP-43 aggregation (Chou *et al.*, 2018), indicating that C9-related NCT and/or NPC defects mediate disease but so far it remains unclear whether TDP-43 pathology is a cause or consequence of it.

**Figure 4:**
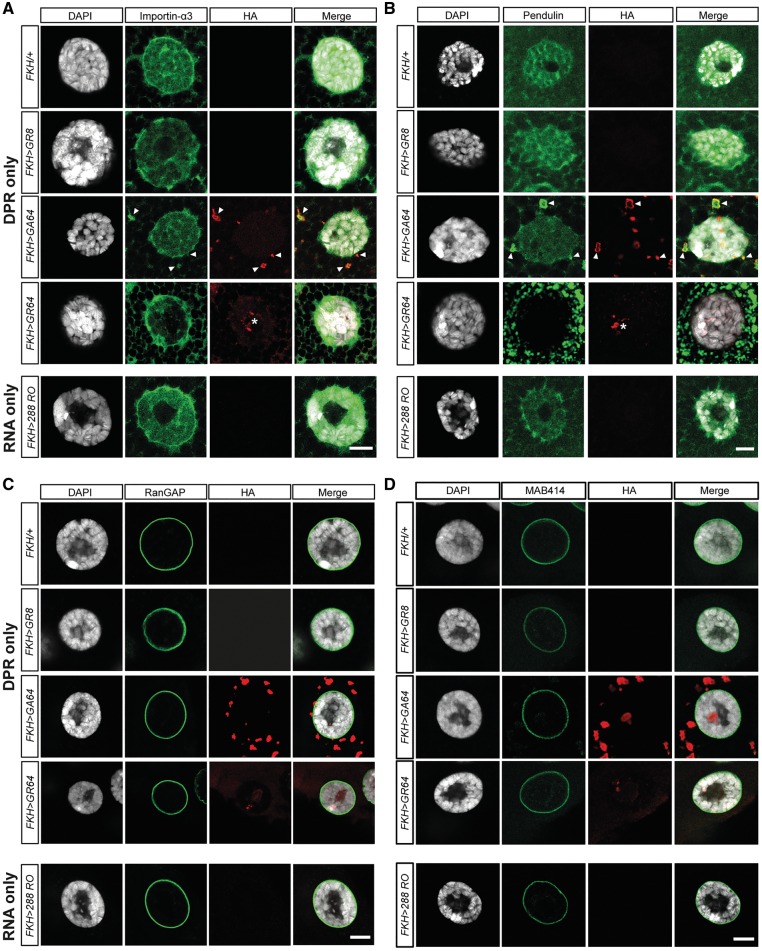
**DPR but not G4C2 RNA accumulation causes karyopherin-α pathology.** Confocal images of larval L3 stage salivary gland cells expressing either non-G4C2, alternative codon-derived DPRs (DPR only) or G4C2 RNA only (288RO) immunolabelled with anti-HA to detect DPR distribution. (**A** and **B**) Additional immunostaining for the karoypherin-αs, importin-α3 (KPNA4) and pendulin (KPNA2) reveals cytoplasmic inclusions that co-localize with poly-GA64 and nuclear depletion in cells expressing poly-GR64, but not in controls or cells expressing 288 G4C2 repeat RNA only. Scale bars = 10 µm. (**C** and **D**) Additional immunolabelling with anti-RanGAP (**C**) and anti-MAB414 (**D**). No changes are seen in localization of these proteins in DPR only and RNA only constructs compared to the controls. Both RanGAP and MAB414 distribution is perinuclear. Scale bars = 10 µm.

To address this question, we first investigated whether accumulation and aggregation of either poly-GA or poly-GR affects the expression and localization of NCT and NPC core components in our C9ALS/FTD models. When expressed in salivary gland cells, we detected already in larval L3 stage perinuclear/cytoplasmic accumulation of KPNA homologues importin-α3 (KPNA4) and pendulin (KPNA2) in flies expressing poly-GA64 or poly-GR64, but not in controls, nor in G4C2 RNA only. Of note, expression of poly-GR64, but not of poly-GA64, also caused nuclear depletion of both importin-α3 and pendulin, whereas expression of poly-GA64 resulted in cytoplasmic inclusions of importin-α3 and pendulin that overlap with poly-GA (Fig. 4A and B).

Conversely, in spite of strong poly-GR toxicity and aggregate formation, no obvious alterations for the NCT component RanGAP were seen (Fig. 4C). Similarly, poly-GR64 expression did not affect localization of dRCC1, the *Drosophila* homologue of Regulator of chromosome condensation 1, a guanine nucleotide exchange factor for Ran GTPase, whereas we detected cytoplasmic dRCC1 inclusions that overlap with poly-GA (Supplementary Fig. 6C). Moreover, when analysing NPC architecture, MAB414 immunolabelling, which recognizes the conserved FG motif (Capelson *et al.*, 2002) in nuclear pore proteins Nup62, Nup153, Nup214 and Nup358, we did not observe any differences between controls, 288RO (G4C2 RNA only), poly-GR64 or poly-GA64 expression in all cases examined (*n* > 10), despite the presence of DPR inclusions (Fig. 4D). Also, Nup50 immunolabelling, which detects a soluble cofactor of importin α / β-mediated cargo transport (Lindsay *et al.*, 2002) across the NPC, did not reveal differences between controls and 288RO, poly-GR64 or poly-GA64 expression (Supplementary Fig. 6D). Collectively, these data suggest that in our *Drosophila* models of C9ALS/FTD, DPR-triggered disease onset correlates with mislocalization and nuclear depletion of KPNA2 and 4, but not with NPC or other NCT deficits.

### Accumulating cytosolic *TBPH* causes KPNA pathology

KPNA2 and KPNA4 are members of the karyopherin-α family and part of the classical nuclear import pathway (Prpar Mihevc *et al.*, 2017). Direct protein–protein interactions between TDP-43 and KPNA2/4 have already been demonstrated (Freibaum *et al.*, 2010; Nishimura *et al.*, 2010; Chou *et al.*, 2018) and dataset of RNA targets of TDP-43 shows that it can bind to *KPNA4* pre-mRNA and to a lesser extent, *KPNA2* (Tollervey *et al.*, 2011). Moreover, KPNAs bind poly-GR/PR (Lee *et al.*, 2016) and are sequestered into cytoplasmic inclusions by β-protein aggregates (Woerner *et al.*, 2016), including fragments of TDP-43. Given that DPR accumulation triggers cytoplasmic mislocalization of TDP-43 (Fig. 2B), we asked whether accumulating cytosolic TDP-43 is itself sufficient to cause KPNA pathology.

To address this question, we first focused on ΔNLS-TBPH that accumulates in the cytoplasm, leads to nuclear TBPH depletion and causes age-related neurodegeneration (Supplementary Fig. 7), thus recapitulating disease-related TDP-43 pathology that also characterizes the majority of C9ALS/FTD cases. Interestingly, ΔNLS-mediated cytoplasmic accumulation of TBPH caused cytosolic mislocalization and nuclear depletion of pendulin/KPNA2, and a nuclear loss of importin-α3/KPNA4. We then investigated whether comparable phenotypes can be observed with full-length wild-type human TDP-43 and the disease-related mutant Q331K. Targeted expression of either *UAS-hTDP-43* or *UAS-Q331K-hTDP-43* (Elden *et al.*, 2010) caused cytosolic mislocalization and reduced nuclear staining of importin-α3/KPNA4 that was not seen in controls or in *TBPH* null mutants (Fig. 3E). In contrast, MAB414 immunolabelling did not reveal any apparent differences between controls, ΔNLS-TBPH and full-length human TDP-43 or its disease-related mutant form Q331K (Fig. 3E). Together these data demonstrate that even in the absence of G4C2 RNA/DPRs, accumulating TDP-43 is sufficient to induce KPNA pathology and suggest that TDP-43 mediated nuclear import deficits initially occur devoid of NPC morphology defects.

### KPNA4 pathology is detectable in both sporadic FTD and C9ALS/FTD human frontal cortex

The results of our experiments signify that DPR-triggered dysfunction of TDP-43 and in turn of KPNAs propagate onset and progression of C9-related neurodegeneration. Consequently, several predictions can be deduced and their validity tested. First and foremost, the reported findings translate into human patients and predict KPNA pathology can be found in C9ALS/FTD but also in sporadic cases devoid of G4C2 repeat expansion. Second, the vicious cycle of DPR-initiated TDP-43 and KPNA pathology predicts that both DPR and TDP-43 inclusions correlate with KPNA pathology, but all three may not be found in the very same cell. To test these predictions, we focused on KPNA4 and examined its expression level and localization in post-mortem human frontal cortex of C9ALS/FTD and sporadic FTD cases with TDP-43 pathology (sFTD-TDP), and compared it to age-matched controls (each *n* = 8).

**Figure 5:**
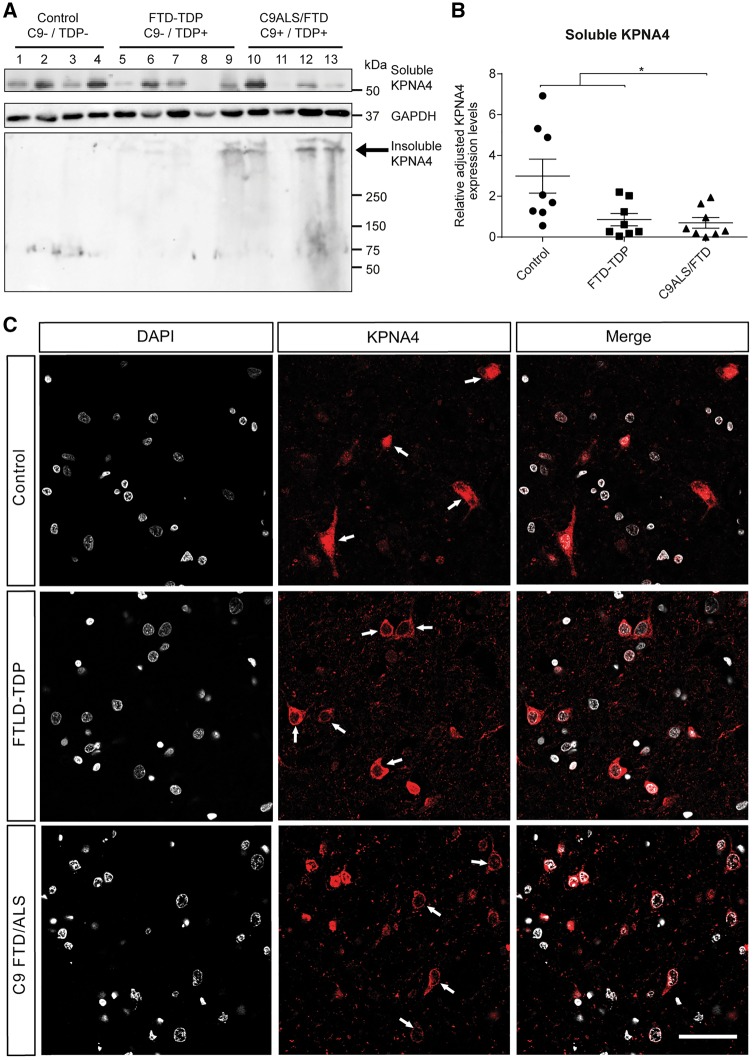
**KPNA4 pathology in both sporadic FTD-TDP and C9ALS/FTD human frontal cortex.** (**A**) Western blot analysis of extracts from post-mortem frontal cortex of patients with sporadic FTD-TDP, C9ALS/FTD and control brain samples. Accumulated KPNA4 is found in the insoluble fraction of sporadic FTD-TDP and C9ALS/FTD samples (black arrow), but not in controls. (**B**) Quantitative analysis of western blotted soluble KPNA4 reveals its downregulation in sporadic FTD-TDP and C9ALS/FTD, but not in control (*n* = 8; *P < 0.03, Bonferroni’s multiple comparison). (**C**) KPNA4 immunostaining in neurons of control cases reveals uniform distribution in both nucleus and cytoplasm (arrows). In sporadic both FTD-TDP and C9ALS/FTD cases, immunolabelling reveals nuclear depletion and cytoplasmic accumulation of KPNA4 (arrows). Scale bar = 50 μm.

Analysis of RIPA and urea protein fractions revealed insoluble aggregates remaining in the wells and lowered expression levels of soluble KPNA4 in both sFTD-TDP and C9ALS/FTD, but not in controls (Fig. 5A and B). KPNA4 immunoreactivity in controls revealed a uniform distribution in neurons with both nuclear and cytoplasmic labelling (Fig. 5C and Supplementary Fig. 8A–D). In contrast, immunohistochemical analysis in both sporadic FTD-TDP and C9ALS/FTD cases identified nuclear depletion of KPNA4 that was further pronounced in C9ORF72 cases (Fig. 5C). However, we also observed nuclear inclusions in both sporadic FTD-TDP and C9ALS/FTD that often were confined to KPNA4-immunoreactive nucleolus within a KPNA4-negative nucleus (Supplementary Fig. 8F and J). Furthermore, neuronal processes consistent with axons and dystrophic neurites were detectable in C9ORF72 cases (Supplementary Fig. 8L).

**Figure 6:**
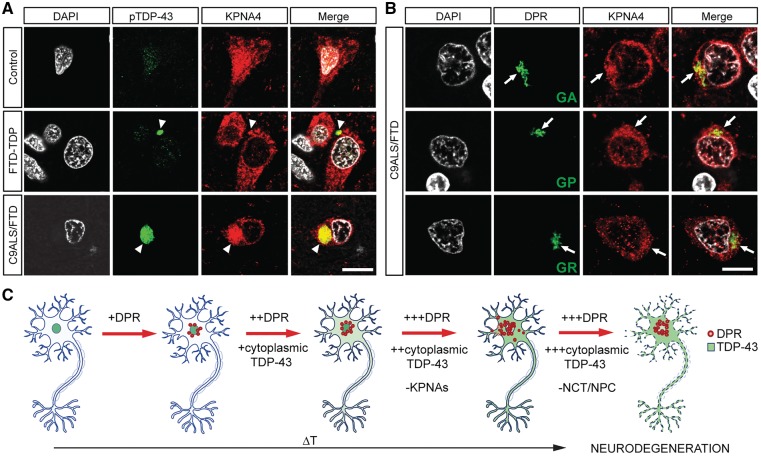
**KPNA4, phospho-TDP and sense DPR pathology in sporadic FTD-TDP and C9ALS/FTD human frontal cortex.** (**A**) KPNA4 immunolabelling in frontal cortex control cases reveals uniform neuronal distribution in both nucleus and cytoplasm (*top row*); immunolabelled phospho-TDP-43 inclusions are detectable in both sporadic FTD-TDP (*middle row*) and C9ALS/FTD (*bottom row*). Note that in both sporadic FTD-TDP and C9ALS/FTD, but not in controls, immunolabelling reveals nuclear depletion and cytoplasmic accumulation of KPNA4 that overlaps with accumulated phospho-TDP-43 (pTDP-43; arrowheads). (**B**) Similarly, in C9ALS/FTD frontal cortex sections, poly-GA, poly-GP and poly-GR inclusions all overlap with clustered cytoplasmic KPNA4 immunolabelling adjacent to nuclear depletion (arrows). (**C**) Proposed mechanism underlying disease formation and progression in C9ALS/FTD. Accumulation of G4C2-derived dipeptide repeat protein causes cytoplasmic mislocalization and accumulation of TDP-43, which in turn leads to nuclear depletion of KPNAs and a vicious cycle of TDP-43 and KPNA dysfunction that together with subsequent further deficits in nucleocytoplasmic transport and the nuclear pore complex, results in neurodegeneration. Scale bars = 10 µm.

The observed KPNA4 pathology overlapped with immunoreactivity against phosphorylated TDP-43 (Fig. 6A, arrowheads). Notably, however, KPNA4 pathology, especially its nuclear depletion, was frequently observed in cells without phospho-TDP-43 labelled inclusions in both sporadic FTD-TDP and C9ORF72 cases (Supplementary Fig. 9, arrows). Moreover, in C9ALS/FTD tissue sections, poly-GA, poly-GP and poly-GR inclusions were detectable that overlapped with KPNA4 pathology (Fig. 6B, arrows), but as was the case for phospho-TDP-43 labelled inclusions, nuclear depletion of KPNA4 was frequently observed in cells devoid of sense DPR inclusions (Supplementary Fig. 10, arrows). These findings identify KPNA4 and TDP-43 pathology as a common denominator of sporadic FTD and C9ALS/FTD, and together with our functional data in *Drosophila* strongly suggest that TDP-43 mislocalization is not just a consequence of defective nuclear import but rather a direct contributor to it.

## Discussion

Our findings reported here provide experimental and pathological evidence for a sequence of events in which DPR-triggered TDP-43 accumulation causes KPNA pathology that precedes *C9orf72*-related neurodegeneration (Fig. 6C). Timing and extent of TDP-43 mislocalization was dependent on levels and identity of DPRs produced. Thus, early cytosolic accumulation and disease onset was observed in the poly-GR producing 38 repeats, and late cytosolic accumulation and disease onset occurred in poly-GP/poly-GA producing lines. Importantly, our C9ALS/FTD models demonstrate that, rather than the length of the G4C2 repeat RNA, toxicity correlates with levels and identity of DPR produced, which is the key stressor causing cytosolic mislocalization of TDP-43 and onset of disease.

A hallmark of TDP-43 proteinopathies is their close correlation between observed clinical phenotypes, degree of neurodegeneration, and regional distribution and severity of TDP-43 pathology (Geser *et al.*, 2009). This close correlation is also observed in the majority of C9ALS/FTD cases, which in addition to TDP-43 pathology are characterized by RNA foci and ubiquitin positive/TDP-43 negative inclusions of G4C2-derived DPRs (Mackenzie *et al.*, 2015). However, neither RNA foci nor DPR inclusions, especially the most abundant poly-GA and poly-GP deposits, correlate with clinical symptoms and neurodegeneration (Mann, 2015). A notable exception has been recently reported in C9ALS cases, which identified cytoplasmic poly-GR inclusions associated with TDP-43 accumulation and site of disease (Saberi *et al.*, 2018). These neuropathological observations suggest a causal link between the hexanucleotide repeat expansion, DPR accumulation and TDP-43 pathology, indicating that TDP-43 dysfunction might be the likely effector of neuronal loss in C9ALS/FTD.

Such a sequence of events is in agreement with the recently discovered NCT and NPC defects in C9ALS/FTD (Freibaum *et al.*, 2015; Jovicic *et al.*, 2015; Zhang *et al.*, 2015; Boeynaems *et al.*, 2016). These studies revealed that targeted genetic manipulation of NCT or NPC genes is able to rescue, at least in part, cellular phenotypes and tissue-specific neurodegeneration in cell and animal models expressing ≥30 G4C2 repeats (Freibaum *et al.*, 2015; Zhang *et al.*, 2015), poly-GR (Lee *et al.*, 2016), or poly-PR (Jovicic *et al.*, 2015; Boeynaems *et al.*, 2016). Yet, TDP-43 pathology has not been reported for NCT or NPC gene mutations or their protein dysfunction. Moreover, several C9ALS/FTD mouse models reported that neurodegeneration only occurred in the presence of TDP-43 pathology (Chew *et al.*, 2015; O’Rourke *et al.*, 2015; Peters *et al.*, 2015; Liu *et al.*, 2016), and clinico-pathological evidence (Proudfoot *et al.*, 2014; Baborie *et al.*, 2015; Vatsavayai *et al.*, 2016) revealed DPR pathology precedes that of TDP-43. Furthermore, NPC deficits have been observed in association with TDP-43 pathology devoid of G4C2 repeat expansion (Chou *et al.*, 2018). This is in agreement with our observation that overexpression of full-length human TDP-43 and its Q331K disease-related mutant, are sufficient to cause importin-α3 nuclear loss and cytoplasmic mislocalization. Wild-type TDP-43 expression has been described as detrimental in flies, even without cytoplasmic accumulation (Hanson *et al.*, 2010). Furthermore, other studies in *Drosophila* (Choksi *et al.*, 2014) have revealed that phosphorylation of TDP-43^Q331K^ leads to formation of SDS-stable oligomers, which may start sequestering surrounding proteins, such as importin-α3. These data suggest that wild-type and mutant TDP-43 overexpression can cause a KPNA phenotype in the absence of DPR pathology, resulting in nuclear depletion and cytoplasmic mislocalization of the KPNAs. However, a limitation when considering the human condition is that some of the conclusions of the study derive from results obtained using constructs that are artificially expressed in fruit flies. Nevertheless, our data indicate that G4C2 RNA and/or DPRs act as initiating stressors causing TDP-43 dysfunction that mediates C9-related neurodegeneration, with NCT and NPC deficits occurring later during disease progression.

In our early onset models of disease, we observed specific KPNA pathology but found no evidence for alterations of other NCT or NPC components such as RanGAP, RCC1 and NPC proteins such as MAB414 and Nup50. These findings are in agreement with a recent study that did not find any evidence for RanGap, lamin B1 and importin β1 pathology in C9ALS cases in which poly-GR pathology was associated with site of disease and TDP-43 pathology (Saberi *et al.*, 2018). We observed a comparable phenotype with targeted cytoplasmic accumulation of *Drosophila* TDP-43, which caused KPNA4 pathology in the absence of NPC pathology (Fig. 3E). Together these data suggest that both poly-GR and TDP-43 dysfunction act through similar, very specific KPNA pathology, but not other NCT or NPC defects, to cause onset of disease. Since targeted expression of poly-GR64 led to rapid accumulation of cytosolic TDP-43 (Fig. 2B), the most parsimonious explanation for these phenotypes are DPR-triggered TDP-43 pathology cause KPNA dysfunction and in turn neurodegeneration. In support of this notion, we observed accumulating cytosolic TBPH not only enhanced DPR production but also toxicity in our models of C9ALS/FTD.

This pathogenic cascade (Fig. 6C) suggests that additional NCT and NPC defects are later events in the progression of disease, a notion consistent with our experimental findings but also with pathological evidence. Thus, we observed KPNA4 pathology in sporadic FTD-TDP cases and C9ALS/FTD human brain that correlated either with phosphorylated (p)TDP-43 or sense DPR pathology, respectively. Notably, in both C9ALS/FTD and FTD-TDP cases we also observed frequent KPNA4 pathology without pTDP-43 inclusions, and in C9ALS/FTD cases, KPNA4 pathology without sense DPR inclusions (Fig. 6A and B). These data suggest, rather than aggregate formation, it is the cytoplasmic accumulation of soluble DPRs and TDP-43, likely as toxic soluble oligomers, that interferes with KPNA function. Consistent with suchlike pathogenic mechanism, we found targeted expression of accumulating cytosolic, but not aggregating TDP-43, caused KPNA pathology and neurodegeneration (Fig. 3E and Supplementary Fig. 7). These findings are consistent with neuropathological studies suggesting that the site of poly-GR toxicity is in the cytoplasm where it overlaps with TDP-43 pathology, rather than the nucleus (Saberi *et al.*, 2018). Indeed, we found targeted poly-GR expression initially accumulated in the cytoplasm, which correlated with onset of TDP-43 and KPNA pathology, rather than its subsequent inclusion formation (Supplementary Fig. 5).

Taken together our findings establish DPR accumulation as a cause of TDP-43 proteinopathy and suggest a vicious feedback cycle for excess cytosolic TDP-43 by which enhanced DPR levels enhance KPNA dysfunction and TDP-43 mislocalization, thereby becoming self-sufficient of the initiating trigger. Unlike G4C2 RNA foci and the vast majority of DPR inclusions (Mackenzie *et al.*, 2015; Mann, 2015; Scaber and Talbot, 2016; Saberi *et al.*, 2018), this pathogenic cascade accords well with distribution of TDP-43 pathology, clinical phenotype and pattern of neurodegeneration, and identifies cytosolic accumulation of non-aggregated TDP-43 as a major culprit of *C9orf72*-related neurodegeneration.

## Supplementary Material

Supplementary Material

## Abbreviations

ALS: amyotrophic lateral sclerosis
DPR: dipeptide-repeat protein
FTD: frontotemporal dementia
NCT: nucleocytoplasmic transport
NLS: nuclear localization signal
NPC: nuclear pore complex
