# Supplementary material for: A feedback loop between dipeptide-repeat protein, TDP-43 and karyopherin-α mediates C9orf72-related neurodegeneration
Source: Brain. 2018 Sep 25;141(10):2908–24. doi: 10.1093/brain/awy241 (PMC6158706; doi:10.1093/brain/awy241)
Supplement: Supplementary Material [file awy241_supplementary_material.pdf]

Supplementary Information

A feedback loop between dipeptide repeat protein, TDP-43 and Karyopherin-*a* mediates *C9ORF72*-related neurodegeneration

Daniel A. Solomon[1,†], Alan Stepto[1,†], Wing Hei Au[1,†], Yoshitsugu Adachi[1], Danielle C. Diaper[1], Rachel Hall[1], Anjeet Rekhi[1], Adel Boudi[1], Jessika C. Bridi[1], Paraskevi Tziortzouda[1], Youn-Bok Lee[1], Bradley Smith[1], Greta Spinelli[1], Jonah Dearlove[1], Dickon M. Humphrey[1], Jean-Marc Gallo[1], Claire Troakes[1], Manolis Fanto[1], Matthias Soller[2], Boris Rogelj[3], Richard B. Parsons[4], Christopher E. Shaw[1],Tibor Hortobágyi[5,6] and Frank Hirth[1,*].

[1]King's College London, Department of Basic and Clinical Neuroscience, Maurice Wohl Clinical Neuroscience Institute, Institute of Psychiatry, Psychology and Neuroscience, London, United Kingdom.
[2]School of Biosciences, College of Life and Environmental Sciences, University of Birmingham, Edgbaston, Birmingham, United Kingdom.
[3]Jozef Stefan Institute, Department of Biotechnology & Biomedical Research Institute BRIS & University of Ljubljana, Faculty of Chemistry and Chemical Technology, Ljubljana, Slovenia.
[4]King's College London, School of Cancer Studies and Pharmaceutical Sciences, London, United Kingdom.
[5]MTA-DE Cerebrovascular and Neurodegenerative Research Group, Departments of Neurology and Neuropathology, University of Debrecen, Debrecen, Hungary
[6]King's College London, Department of Old Age Psychiatry, Institute of Psychiatry, Psychology and Neuroscience, London, United Kingdom.

[†]These authors contributed equally to this work.

*Correspondence to:
Dr. Frank Hirth, King's College London, Department of Basic and Clinical Neuroscience, Maurice Wohl Clinical Neuroscience Institute, Institute of Psychiatry, Psychology and Neuroscience, Cutcombe Road, SE5 9RX, London, United Kingdom; Tel: ++44 20 7848 0786; email: Frank.Hirth@kcl.ac.uk

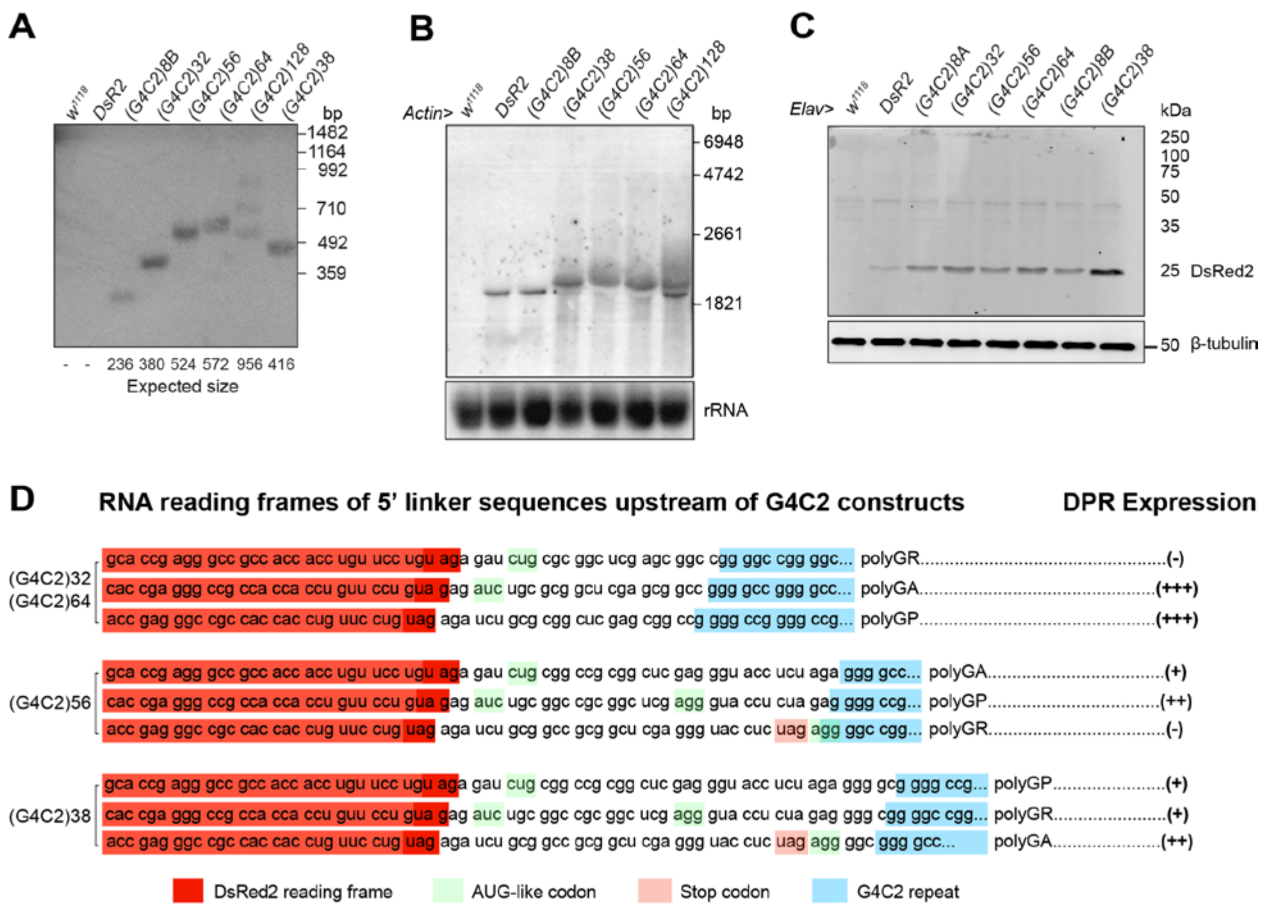

Supplementary Fig. 1 G4C2 RNA expression and derived RAN translated DPRs depend on 5' linker sequences. (**A**) Southern blot of controls and indicated G4C2 repeats of different lengths. (**B)** Northern blot using a probe against DsRed2 mRNA indicates high molecular weight RNA for G4C2 repeats. (**C**) Western blot analysis of day 5 head extracts showing expected ~26 kDa DsRed2 band in all G4C2 constructs, but not in negative control (*Elav/white*), indicating no read-through translation of *DsRed2* stop codon into G4C2 repeats. (**D**) Schematic of constructs with different lengths of uninterrupted G4C2 repeats cloned into 3' UTR of disease-unrelated marker gene *DsRed2* (in red); variable linker sequences between DsRed2 stop codon and start of G4C2 repeats are indicated, as are AUG-like codons and DPR produced. Expression levels (+, low; ++, moderate; +++ high) of RAN-translated DPR produced by specific G4C2 repeat construct.

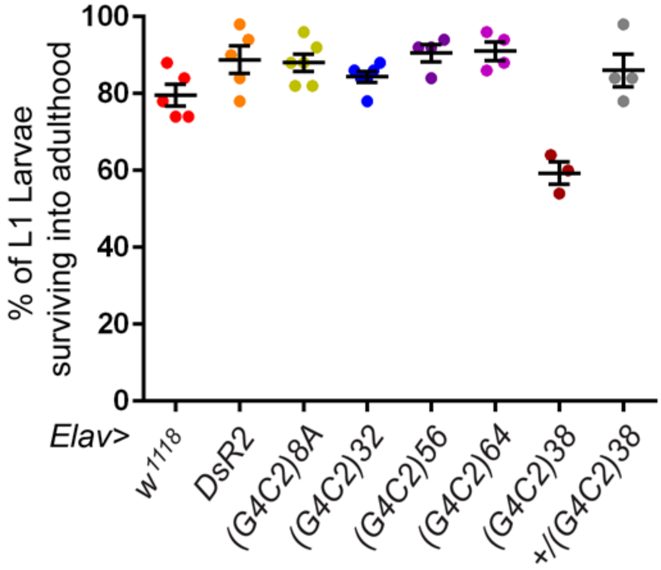

Supplementary Fig. 2 Impaired larval survival following pan-neuronal expression of G4C2 repeats is observed only in flies expressing 38 repeats. L1 larvae expressing G4C2 repeats in a pan-neuronal pattern were monitored throughout development. Significant difference in the number of larvae successfully developing into adults was reported dependent on genotype (One-way ANOVA, F=10.33, p<0.0001). Post-hoc analysis with the Bonferroni-Holm correction for multiple testing found significant impairment in larval survival in larvae expressing 38 repeats relative to all other G4C2 repeat expressing lines (p<0.0001), $Elav^{C155}$>*DsR2* control (p<0.0001) and $Elav^{C155}/w^{1118}$ control (p<0.0001). Importantly, survival in flies expressing 38 repeats was also impaired relative to the *+/(G4C2)38* UAS control (p<0.0001), confirming that this effect was not due to the genomic insertion in the UAS line. Interestingly, larval survival in flies expressing 8A, 32, 56 and 64 repeats did not significantly differ from each other or $Elav^{C155}/w^{1118}$ or $Elav^{C155}$>*DsR2* controls. Each data point represents the percentage of larvae surviving to adulthood from an experimental pool of 50 L1 larvae (n=3-6, error bars represent SEM).

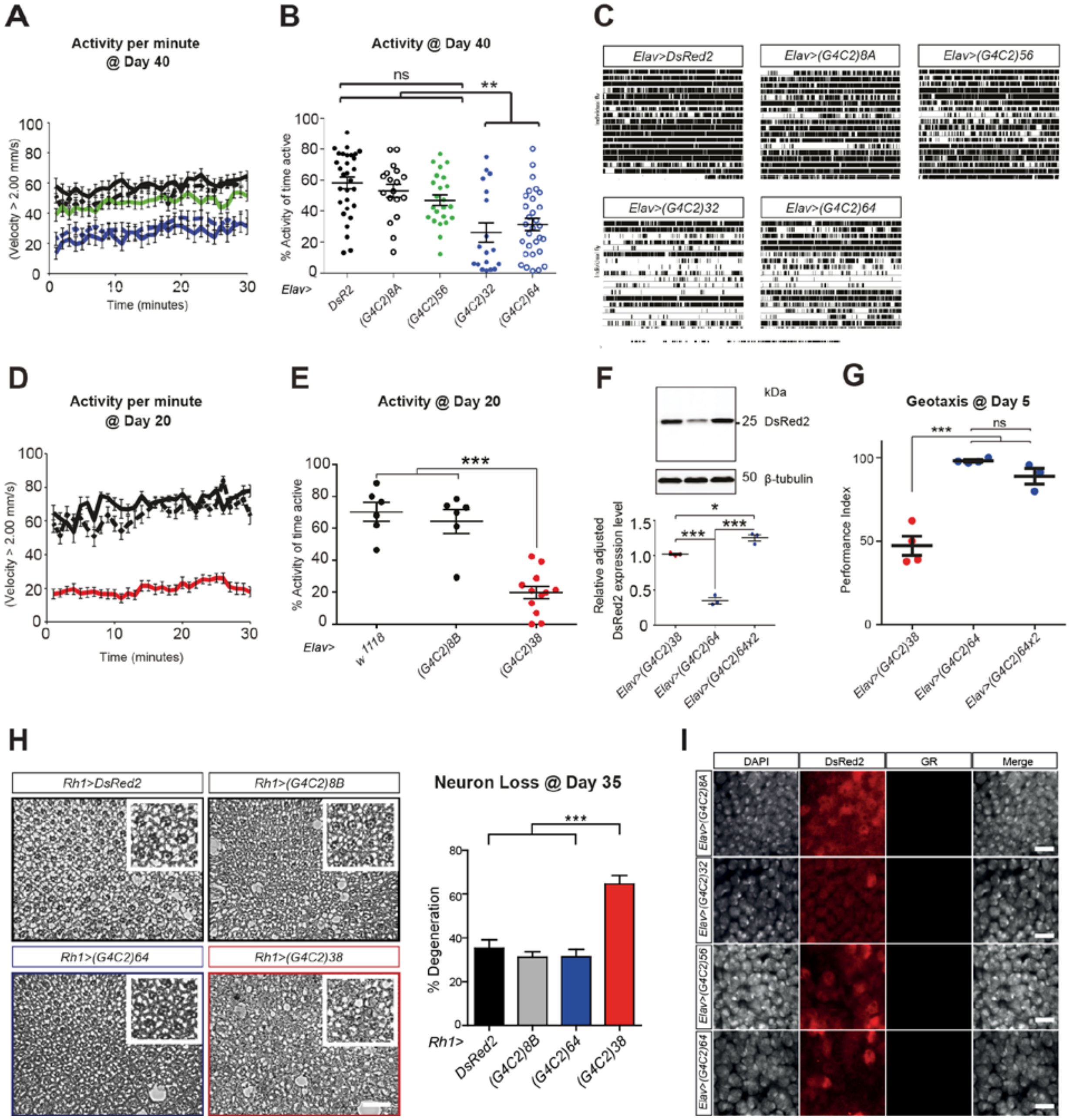

Supplementary Fig. 3 Pan-neuronal expression of G4C2 repeats induce motor impairment and neurodegeneration that vary in age of onset and severity. (A) Open field behavioral activity and (**B)** mean activity at day 40 of flies expressing different G4C2 repeats that lead to different levels of poly-GA and poly-GP produced (compare with Fig. 1B,C and Table 1). Percentage of time active plotted for each fly in each genotype showing that flies expressing 32 and 64 repeats, were significantly less active than control and flies expressing 8 or 56 repeats. **p<0.01; data points are mean % activity for individual flies with overall mean and SEM (n=18-30). (**C)** Raster plots demonstrating activity of individual flies over a 30 minute period. White bars represent periods of inactivity whereas black bars represent bouts of activity. Flies with 32 or 64 G4C2 repeats that express high levels of poly-GP and poly-GA were less active compared to controls and flies with 56 G4C2 repeats that expresses much lower levels of poly-GP and poly-GA (compare with Fig. S1 and Table 1). (**D, E)** Open field behavioral activity at day 20; percentage of time active plotted for each fly in each genotype showing that flies expressing 38 repeats were significantly less active than controls; ***p<0.001, data points are mean % activity for individual flies with overall mean and

SEM (n=6-12). Collectively, flies expressing 38 repeats were less active across the entire 30 min period, than controls expressing 8 or 0 repeats. (**F)** Quantitative western blot analysis of DsRed2 revealed that flies expressing 38 repeats have a significantly higher expression level than flies expressing 64 repeats ($p<0.001$). However, flies expressing 2x 64 repeats produced a significantly higher level of DsRed2 expression than those expressing 38 repeats; *$p<0.05$; ***$p<0.001$, mean and SEM shown for each genotype (n=3). (**G)** Geotaxis climbing performance of day 5 flies with pan-neuronal expression of 64 and 2x64 G4C2 repeats compared to flies expressing 38 G4C2 repeats. Note that motor behavior phenotype does not correlate with expression level but with levels and identity of DPRs produced (compare with Supplementary Fig. 1 and Table 1). ***$p<0.001$; mean shown with SEM (n=3-4). (**H)** Semi-thin retinal sections of 35 days old flies over-expressing *DsRed2* (n=5), (G4C2)8B (n=6), (G4C2)38 (n=6) or (G4C2)64 (n=5), under the control of Rhodopsin1 promotor (*Rh1>*). Higher magnification of the retina structure is displayed on the top right inset; histograms shows quantitative analysis of percentage photoreceptor loss, ***$p<0.0001$ with mean and SEM shown; scale bar, 20µm**. (I)** Confocal images of nervous system of *Elav-Gal4* mediated pan-neuronal expression of controls and indicated G4C2 repeats. Immunohistochemistry of 5-day old adult brains shows no poly-GR detectable in controls and flies expressing 32, 56 or 64 G4C2 repeats. Scale bars, 10µm.

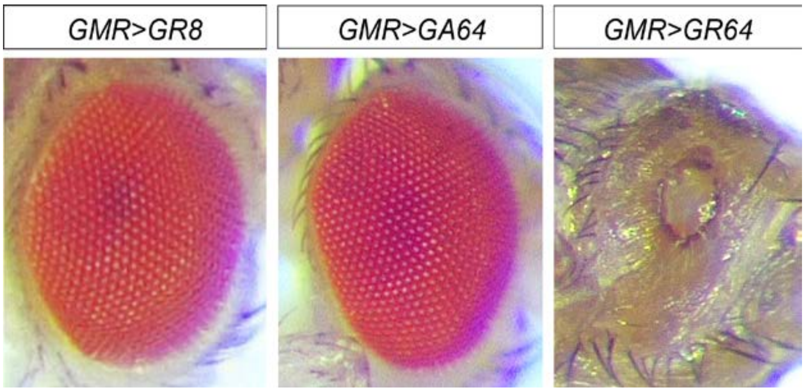

Supplementary Fig. 4 Expression of arginine-rich poly-GR64, but not poly-GA64 or poly-GR8 control, causes degenerative eye phenotype. Day 2 old adult *Drosophila* eyes of *GMR-Gal4* targeted expression of poly-GR8 control (left), poly-GA64 (middle) or poly-GR64. Note the almost absent eye in *GMR>GR64*.

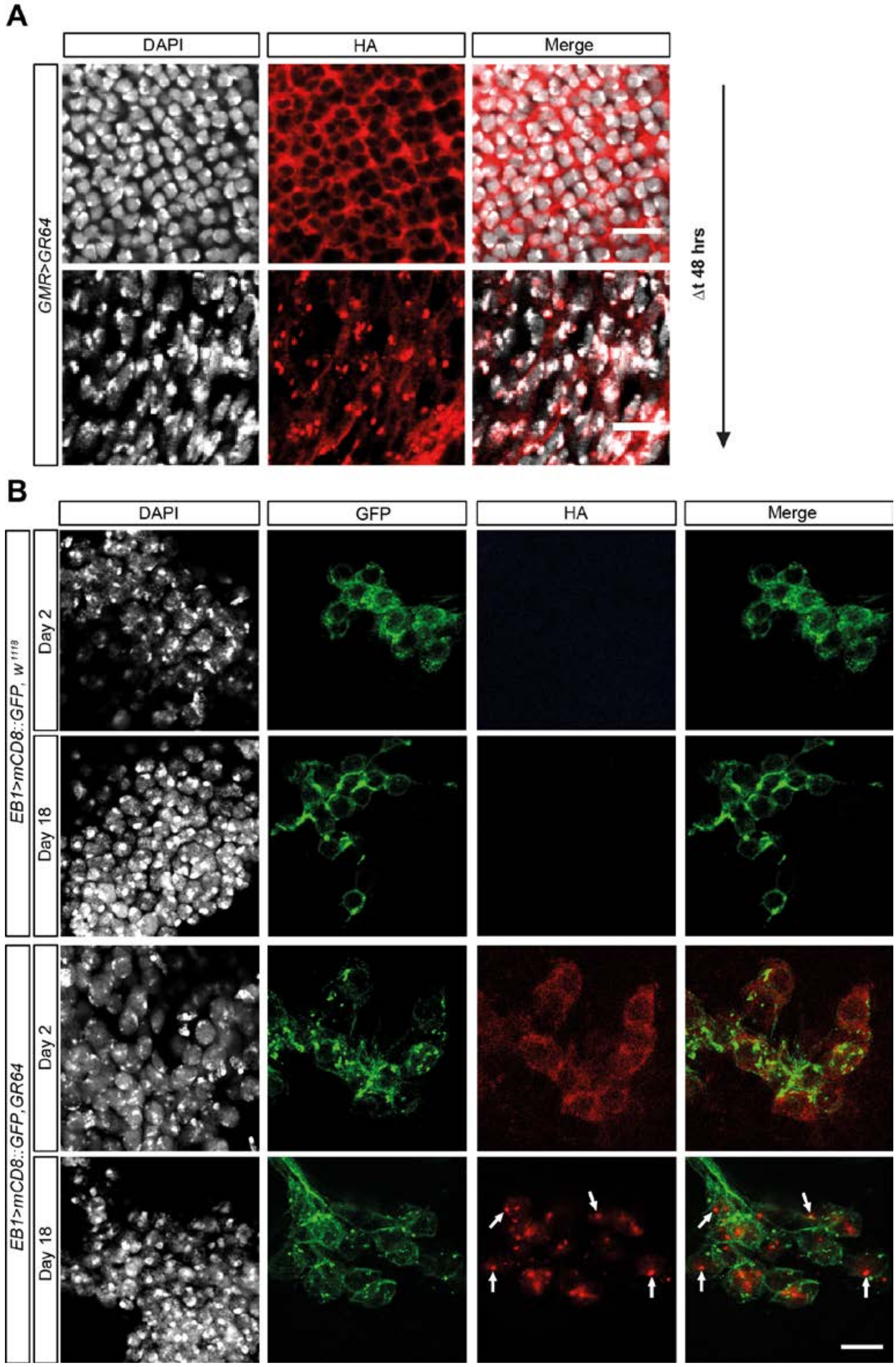

Supplementary Fig. 5 Accumulation of poly-GR changes over time from diffuse cytoplasmic to predominantly aggregated distribution. (A) Confocal images of L3 and pupal eye discs of *GMR>GR64* flies immunolabeled for HA and DAPI, indicating poly-GR distribution. Note, the localization of HA/poly-GR initially reveals diffuse cytoplasmic distribution and within 48 hours (L3 to pupal transition) also shows nuclear inclusions. (**B)** Confocal images of adult central brain neurons using *EB1-Gal4* driver at day 2 and day 18 expressing *UAS-mCD8::GFP* either alone or in conjunction with *UAS-poly-GR64*. Immunolabeling for HA reveals diffuse cytoplasmic and perinuclear distribution of poly-GR by day 2 and by day 18 also identifies nuclear and cytoplasmic inclusions (arrows). Scale bars, 10µm.

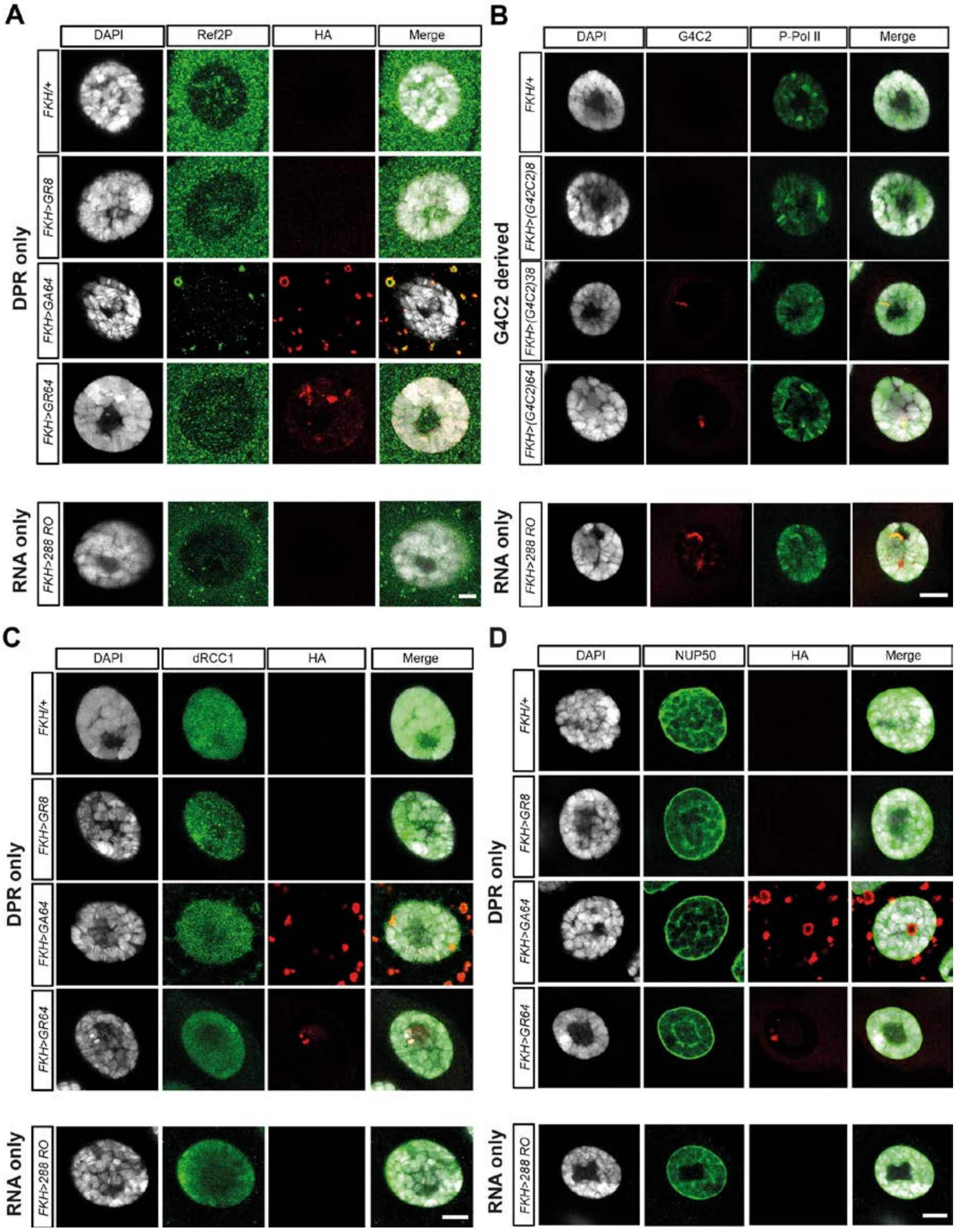

Supplementary Fig. 6 GA and GR but not G4C2 RNA accumulation cause foci formation. Confocal images of salivary gland cells expressing either G4C2 RNA only (288RO), or G4C2 repeats or non-G4C2, alternative codon derived DPRs. (**A**) *Drosophila* Ref2P, the *Drosophila* homologue of p62, is sequestered into poly-GA64 inclusions, but not into nuclear inclusions of poly-GR64 nor controls or cells expressing 288 G4C2 repeat RNA only. (**B**) Fluorescence *in situ* hybridization of G4C2 probe together with immunostaining for phospho RNA Polymerase II (p-Pol-II) identifies co-localization at site of transcription in cells expressing 288 G4C2 repeat RNA only, as well as for 38 and 64 G4C2 repeats, but additional RNA foci non-overlapping with p-Pol-II only for 288RO. (**C**) Immunostaining for dRCC1 and (**D**), nucleoporin NUP50, for which no changes in nucleocytoplasmic localisation and nuclear pore morphology can be seen in all indicated conditions, even in the presence of poly-GA64 aggregates. Scale bars, 10µm.

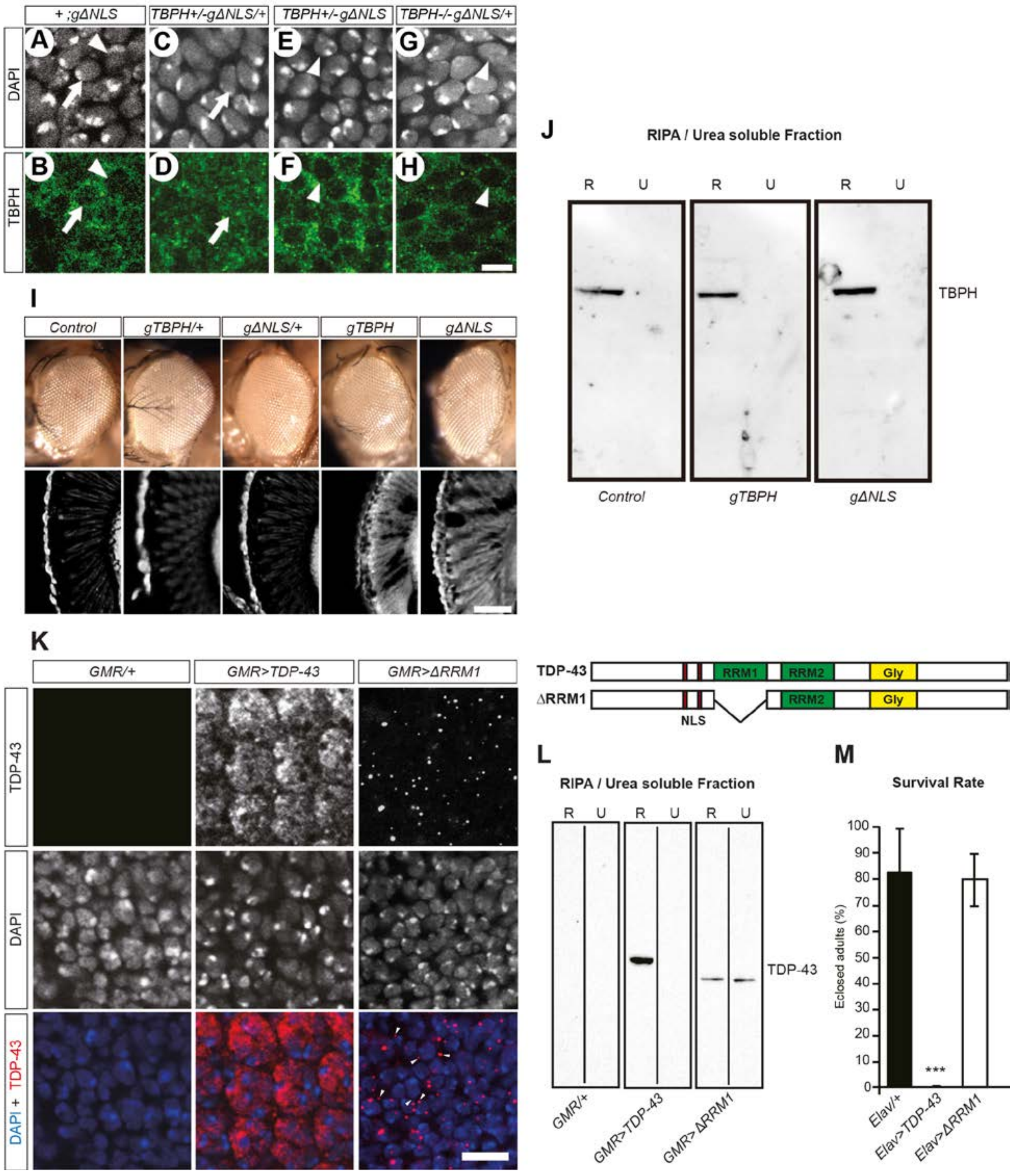

Supplementary Fig. 7 Accumulating cytosolic, but not aggregating TDP-43 causes neurodegeneration. (**A-H)** Confocal images of third instar larval eye discs immunolabeled with anti-TBPH. Compared to controls (**A, B**), flies heterozygous for a deletion of *TBPH* that also express one copy of a genomic construct ΔNLS-TBPH (gΔNLS), reveal (**C, D**, arrow) diffuse cytoplasmic and nuclear TBPH localization; however in heterozygous *TBPH* mutants with two copies of ΔNLS-TBPH, nuclear clearance of TBPH is detectable (**E, F**, arrowhead) which is also seen (**G, H**) in homozygous *TBPH* null mutants expressing one copy of ΔNLS-TBPH. (**I)** External and internal adult eye morphology of control w^{1118}, heterozygous g*TBPH* (*gTBPH/+*), heterozygous ΔNLS-TBPH (*gΔNLS/+*), homozygous g*TBPH* (*gTBPH*), and ΔNLS-TBPH homozygous (*gΔNLS*) are shown. Note internal degeneration seen in flies expressing two copies of either of the genomic constructs (g*TBPH* and gΔNLS). (**J)** Western blot analysis of RIPA soluble (R) and Urea soluble

(U) fractions from head extracts of 5 day old control flies or homozygous for g*TBPH* or ΔNLS-TBPH homozygous (*gΔNLS*). Note that in all cases, anti-TBPH immunolabeling is only detectable in soluble RIPA fraction. (**K)** (right) schematic showing protein domains of human wild-type TDP-43 and mutant TDP-43 devoid of RNA recognition motif 1 *(*ΔRRM1*)*. Left, confocal images of third instar larval eye discs immunolabeled with antibody against human TDP-43 and DAPI. Compared to control (*GMR/+*), GMR-Gal4 specific expression of *ΔRRM1-TDP-43* but not of wild-type full length *TDP-43* leads to inclusions that are found both in the cytoplasm (arrowheads) and the nucleus. (**L)** Western blot analysis of RIPA soluble (R) and Urea soluble (U) fractions from head extracts of 5 day old control flies (GMR/+) or flies expressing either *ΔRRM1-TDP-43* or wild-type full length *TDP-43.* Note that anti-TDP-43 immunolabeling reveals a 43 kDa TDP-43 band only in RIPA soluble fraction of *GMR>TDP-43*. TDP-43*(ΔRRM1)* protein is detected in both RIPA and urea fraction, identifying urea soluble aggregates; note that molecular size is smaller than TDP-43 due to RRM1 deletion. (**M)** Survival rate analysis. Histogram depicts percentage of animals that survived transition from development to adulthood with Elav-Gal4 specific, pan-neuronal expression of human wild-type TDP-43 or *ΔRRM1-TDP-43*; mean and 95% confidence interval are shown. Note that neuronal expression of full length TDP-43 is lethal whereas neuronal expression of *ΔRRM1-TDP-43* leads to survival rate similar to control (*Elav/+*), even in the presence of cytoplasmic and nuclear aggregates (compare to B). ***p<0.001. Scale bars, 50 µm.

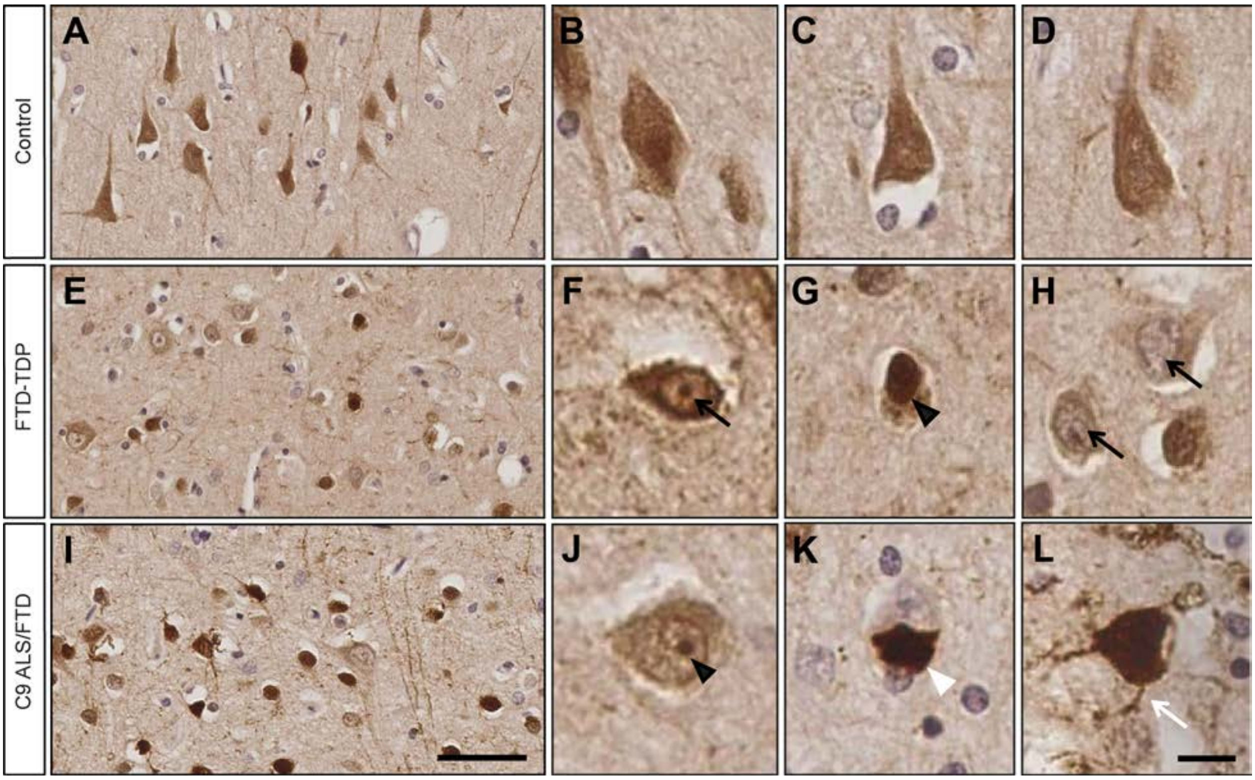

Supplementary Fig. 8 Nuclear depletion and cytoplasmic accumulation of KPNA4 in sporadic FTD-TDP and C9ALS/FTD human frontal cortex. (**A-D**) DAB immunolabelling in controls shows a uniform KPNA4 distribution in neurons with both nuclear and cytoplasmic localisation. (**E-H)** In sporadic FTD-TDP cases, cells lacking nuclear KPNA4 immunolabelling show nucleolar immunoreactivity (**F**). Cells with increased nuclear staining show decreased cytoplasmic labelling (**G**, black arrowhead), whereas cells with nuclear depletion show preserved or mildly increased cytoplasmic immunolabelling (**H**, black arrows). (**I-L)** In C9ALS/FTD cases, KPNA4 DAB immunolabelling in the nucleolus is also observed compared to sporadic FTD-TDP and control cases (**J**). Numerous cells with absent nuclear KPNA4 immunostaining display immunoreactive cytoplasmic inclusions (**K,** white arrowhead) and also immunolabelled neuronal cell processes indicative of dystrophic neurites (**L,** white arrow). Scale bars, 50µm (I), 10µm (L).

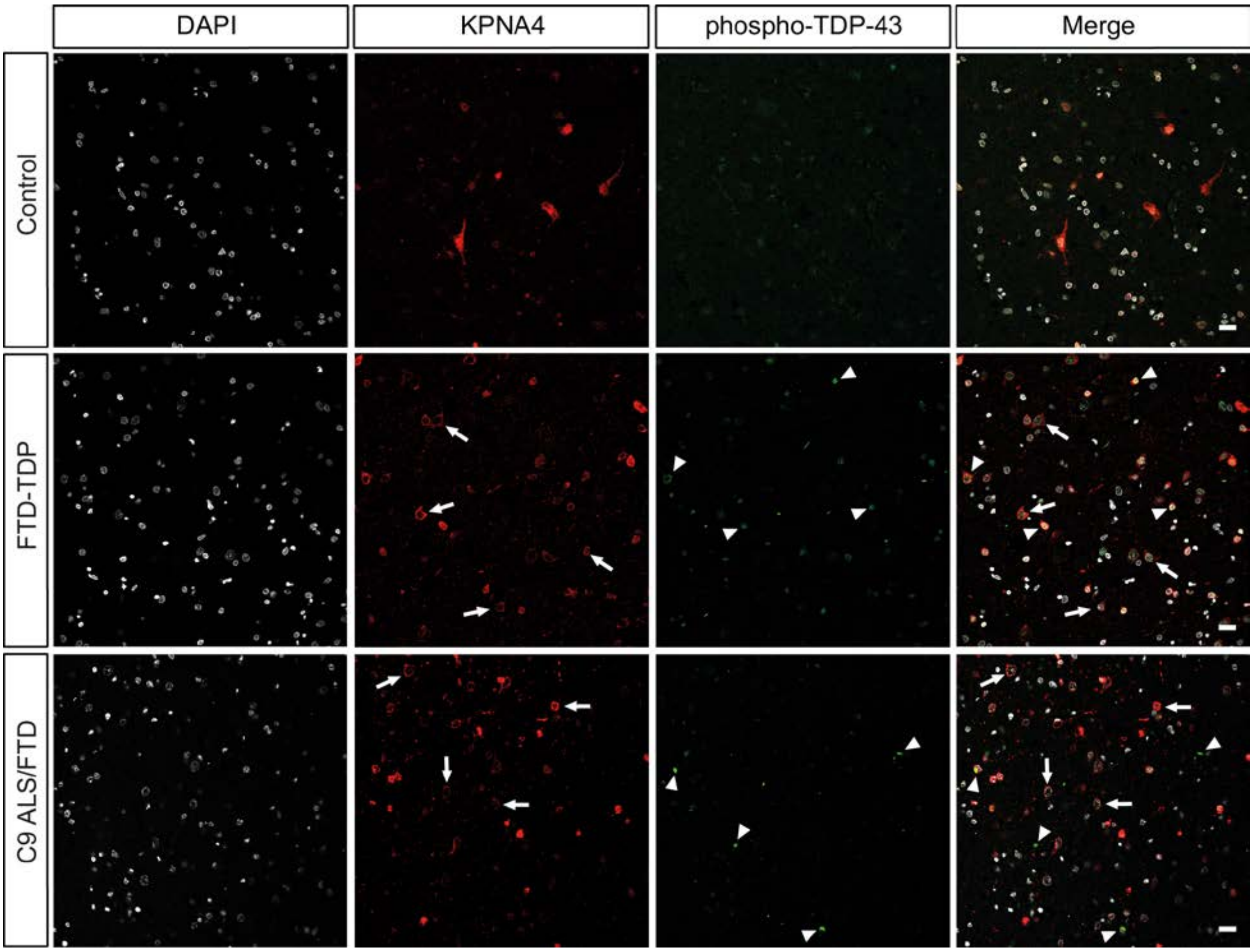

Supplementary Fig. 9 KPNA4 pathology and phospho TDP-43 inclusions in sporadic FTD-TDP and C9ALS/FTD human frontal cortex. Frontal cortex fixed tissue sections immunolabeled for KPNA4, phosphorylated TDP-43 and DAPI reveal TDP-43 inclusions (white arrowheads) and nuclear depletion of KPNA4 in both sporadic FTD-TDP and C9ALS/FTD (white arrows), but not in controls. Note that in both sporadic FTD-TDP and C9ALS/FTD, nuclear depletion of KPNA4 is detectable also in cells without TDP-43 inclusions. Scale bar, 50μm.

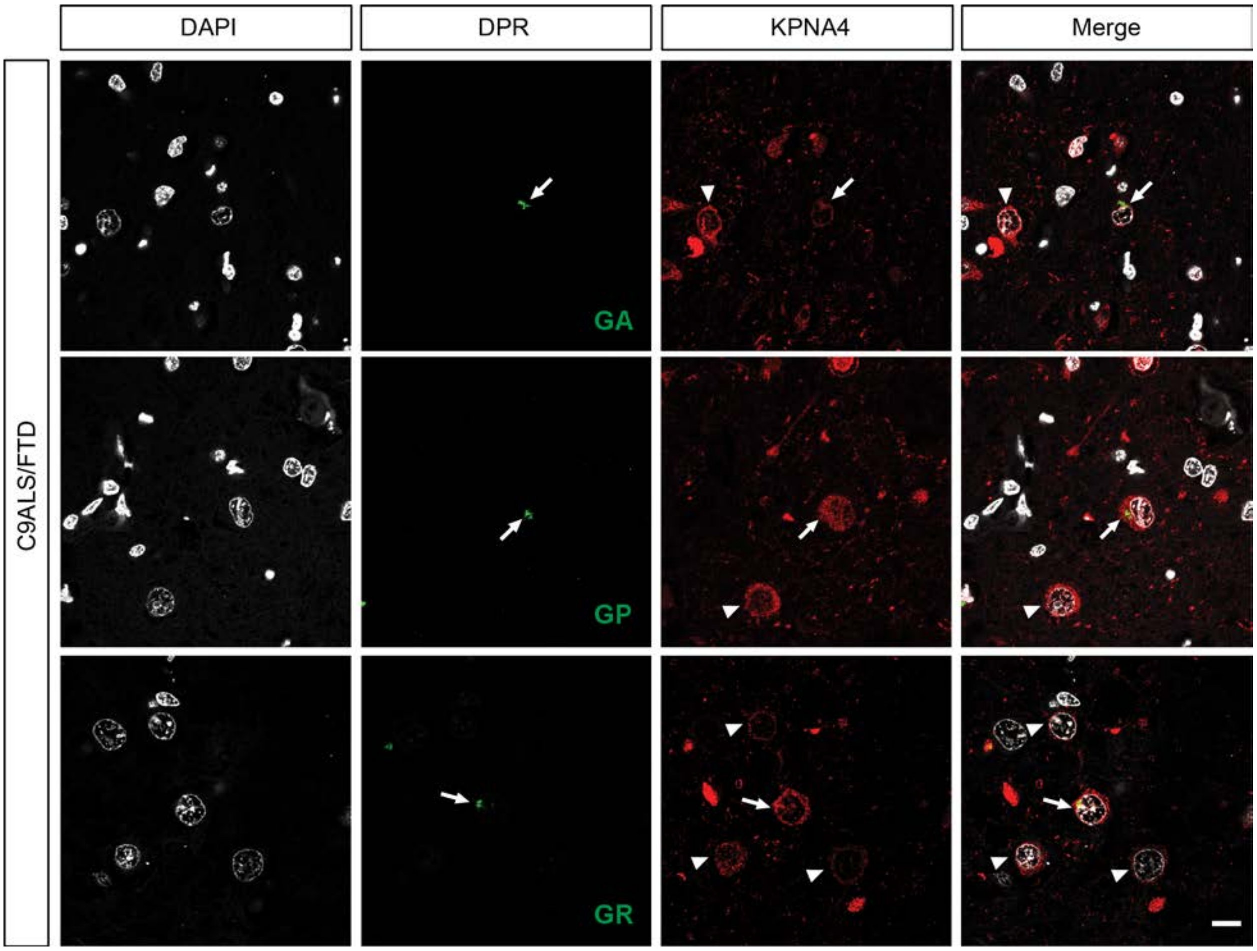

Supplementary Fig. 10 KPNA4 pathology and sense DPR inclusions in C9ALS/FTD human frontal cortex. Frontal cortex fixed tissue sections immunolabeled for KPNA4, sense DPRs and DAPI reveal respective DPR inclusions (white arrowheads) and nuclear depletion of KPNA4 in C9ALS/FTD (white arrows). Note that in C9ALS/FTD, nuclear depletion of KPNA4 is detectable also in cells without detectable DPR inclusions. Scale bar, 50µm.

Detailed Materials and Methods

Fly stocks and husbandry. Fly stocks were maintained at 25°C on standard cornmeal food, unless for aging experiments where flies were maintained on 15% sugar/yeast medium. The following strains were used derived from the Bloomington stock centre unless stated otherwise: *Oregon R*; *w^{1118}*; *Actin-Gal4*; *GMR-Gal4*; *$Elav^{C155}$-Gal4*; *$Elav^{C155}$;UAS-mCD8-GFP; Gal80ts* (a gift from L. Partridge); *Fkh-Gal4* (a gift from E. Baehreke); *UAS-mCD8::GFP*; *UAS-hTDP-43; UAS-Q331K-TDP-43* (Elden *et al.*, 2010); *UAS- ΔRRM1-TDP-43* (Ihara *et al.*, 2013; a gift from T. Iwatsubo); and *gTBPH* (Diaper *et al.*, 2013).

Generation of transgenic flies. The following UAS-attB constructs were generated during the present study: *UAS-DsRed2, UAS-DsRed2-(G4C2)8A, UAS-DsRed2-(G4C2)32, UAS-DsRed2-(G4C2)56, UAS-DsRed2-(G4C2)64, UAS-DsRed2-(G4C2)128B, UAS-DsRed2-(G4C2)8B, UAS-DsRed2-(G4C2)38, UAS-GA8, UAS-GA64, UAS-GR8, UAS-GR64, UAS-PR8, UAS-PR64, UAS-PA8, UAS-PA64.* G4C2 constructs were established that contained DsRed2 upstream of either (G4C2)8, (G4C2)38 or (G4C2)56. In order to generate plasmids containing longer repeat lengths, a further plasmid containing (G4C2)4 repeats within the 3'UTR was generated. Two copies of this G4C2 plasmid were then differentially digested such that subsequent ligation created a plasmid containing a doubled G4C2 repeat sequence. This strategy was employed to generate plasmids containing 8, 16, 32, 64 and 128 G4C2 repeats. OneShot TOP10 chemically-competent bacterial cells (Invitrogen) were then transformed with plasmid DNA according to the manufacturer's instructions. Plasmids were purified using the Qiagen Plasmid Midi Kit (Qiagen). Size of G4C2 repeat in these plasmids was confirmed by gel electrophoresis. The sequence of plasmid DNA from strains containing bands of the expected size was then confirmed by Sanger sequencing (Source Bioscience, UK). The sequencing primers were CTACTACTACGTGGACGC (forward) and TCCATAGGTTGGAATCTAAA (reverse). DNA from clones containing the correct number of G4C2 repeats was then sent to Bestgene, USA for generation of transgenic flies. The presence and size of G4C2 DNA within the fly genome was confirmed by southern blotting, which produced expected results for all UAS lines except those containing 128 repeats, which displayed repeat instability (Supplementary Figure 1A) and were thus excluded from further analysis. DPR only constructs using alternative codon sequences coding for poly-GA, poly-GR, poly-PA and poly-PR were designed for 8aa and 64aa. Plasmid DNA was synthesized by GeneArt (Life Technologies) and sent to BestGene (USA) for generation of transgenic flies. Constructs were integrated into the genome at a common insertion site through φC31-mediated site-specific integration at site ZH-

86FB for UAS-DsRed2-(G4C2) constructs and attP40 for alternative codon DPR only constructs. Genomic insertion was carried out by Cambridge transgenic fly facility (Cambridge University, UK) and Bestgene (USA). Construction of UAS flies carrying full length human TDP-43 (*UAS-hTDP-43*) and of flies carrying genomic construct *ΔNLS-TBPH* was carried out as previously described (Diaper *et al.,* 2013). Nuclear localization signal (NLS) mutant (gΔNLS) was generated by mutation containing PCR.

Reverse transcription PCR. RNA was extracted from homogenized fly heads. RNA was converted to cDNA via mouse megalovirus reverse transcriptase (M-MLV RT; Promega) and random hexamer oligonucleotide primers (Fermentas) following the manufacturer's instructions. Samples were incubated at 37°C for 1 hour and 70°C for 15 minutes. The following primers were used to look at *TBPH* mRNA levels: forward primer: 5′- ATCTTGGATGGCTCAGAACG-3′, reverse primer: 5′-GTCGGTCTTTATTCCGTTGG-3′. RPL32 was used as a loading control with the following primers: forward primer: 5′-CGCCGCTTCAAGGGACAGTATC-3′, reverse primer: 5′-CGACAATCTCCTTGCGCTTCTT-3′.

Southern blotting. Genomic DNA extraction from 30 male flies per genotype was extracted using the Wizard Genomic DNA kit (Promega). Double digestion reactions (DNA from each genotype, Alul (NEB) and Ddel (NEB)) were incubated at 37°C overnight. Digested DNA was run for 6 hours at 40V. After depurinating the gel (0.25M HCl), the gel was then neutralized and alkaline denatured (0.4M NaOH). The DNA was transferred onto Hybond N+ membrane paper (GE Healthcare) by capillary action overnight. Following transfer, the membrane was exposed to UV light for 1 minute, washed with 20ml DIG Easy Hyb solution (Roche) for 1 hour at 65°C and a further 2 hours at 48°C. 10ng of DIG-(C4G2)8-DIG labeled oligonucleotide probe (Integrated DNA Technologies) added to pre-heated DIG Easy Hyb solution and incubated with the membrane at 48-49°C overnight. The hybridization solution was removed and sequential washing of the membrane was conducted using the DIG wash and block buffer set (Roche), according to the manufacturer’s instructions. The anti-DIG antibody (Roche) was added to blocking solution and exposed to the membrane for 30 minutes at room temperature. The membrane was washed 3x 15 minutes with 1x washing buffer (Roche) at room temperature, followed by 1x detection buffer (Roche) for 5 minutes before being exposed to CSPD (Life Technologies) at a concentration of 1:100 in 1x detection buffer, for 2 minutes. The membranes were exposed to X-ray films (GE Healthcare), which were developed and fixed with an automated processor (Konica Minolta, SRX-101A).

Northern blotting. RNA extraction from around 70 L3 larvae was carried out using the RNeasy Lipid Tissue kit (Qiagen), according to the manufacturer's instructions. Samples were heated to 50°C for 30 minutes before being transferred to ice for 2 minutes. The gel was run for 4 hours at 75V and RNA was transferred onto N+ hybond membrane paper (GE Healthcare) for 19 hours. Probe hybridization and detection following northern blotting followed a similar protocol as for Southern blotting. Generation of a DIG-labelled probe for detection of DsRed2 RNA was generated using the PCR DIG probe synthesis kit, according to the manufacturer's instructions (Roche). Primers specifically targeting a coding region of DsRed2 were used (forward: GTGATGCAGAAGAAGACCAT and reverse: CTTGGCCATGTAGATAGACT).

Generation of polyclonal and monoclonal poly-GP antibodies. Two anti-GP antibodies were generated during this project. To generate rabbit polyclonal anti-polyGP, a custom made peptide sequence – GPGPGPGPGPGPGPGPGPGPGPGPGPGPGP (GPx15) - was fused to the C-terminus of maltose-binding protein. Two rabbits were immunized with the fusion protein and the resulting serum was purified with GST-fusion proteins containing $(GP)_{15}$ at the C-terminus. Peptide generation and immunization was carried out by Eurogentec. Mouse monoclonal anti-polyGP (clone 2C20) was generated by immunization of mice with the custom-made peptide sequence - GPGPGPGPGPGPGPGPGPGP (GPx10). This work was conducted by Abmart.

Western blotting. For *Drosophila* samples, heads from mated female flies were homogenized in RIPA buffer. Total protein concentration was measured using the BCA kit (Thermoscientific), according to the manufacturer's instructions (Thermo Scientific Pierce). Sample was mixed with 5X Laemmli sample buffer, boiled for 5 minutes, separated on an SDS-PAGE gel and transferred to the nitrocellulose membrane (Pall). The membrane was blocked (1:1 Odyssey blocking buffer and TBS), probed with primary antibody solution and left overnight at 4°C. Membranes were then washed in TBS-T and incubated with secondary antibody for 1 hour. Membranes were further washed in TBS-T and TBS before imaging. The signal was visualized with either chemiluminescent substrate (GE Healthcare) or by using an Odyssey Fc (Li-Cor). For detection of insoluble protein, samples were lysed in RIPA buffer and then spun at 16,000xg for 10 minutes, after which the supernatants were separated from the pellets and prepared for western blot. The pellets were washed three times with RIPA buffer and then resuspended in urea buffer (7M urea, 2M thiourea, 4% CHAPS, 30 mM Tris). Samples were spun at 16,000xg for 10 minutes, after which the supernatant was prepared for western blot. The following primary antibodies and dilutions were used: rabbit anti-DsRed2 (1:1000, Clontech); mouse monoclonal anti-GP (1:1000, this work); mouse monoclonal anti-GA (clone 5E9) (1:1000, Merck Millipore, MABN889); rabbit polyclonal anti-

TBPH (1:2000) (Diaper *et al.*, 2013); mouse anti-TARDBP 2E2-D3 (1:100, Abnova); rabbit anti-beta actin polyclonal (1:1000, Abcam); mouse anti-beta tubulin monoclonal (1:600, DSHB); goat anti-KPNA4 (1:750, Abcam); rabbit anti-GAPDH (1:5000; Cell Signaling Technology). The following secondary antibodies and dilutions were used: Polyclonal goat anti-rabbit IgG (H&L) conjugated IRDye800 (Rockland immunochemicals) used at 1:10000 and polyclonal goat anti-mouse IgG (H&L) conjugated Alexa Fluor 680 (Thermo Fisher) used at 1:10000.

Dot blotting for DPR detection. Dot blots were used to detect DPRs using the protocol described previously (Mizielinska *et al.*, 2014). Primary and secondary antibodies were diluted in OBB with 0.1% (v/v) tween-20 (Sigma-Aldrich) as listed; rabbit anti-GR (1:1000, Mann *et al.*, 2013), rabbit anti-GR (1:1000, Gendron *et al.*, 2013), rabbit anti-PR (1:1000, Gendron *et al.*, 2013); rabbit anti-PA (1:1000, Gendron *et al.*, 2013) anti-beta tubulin mouse (1:300, DSHB). Secondary antibodies used were polyclonal goat anti-rabbit IgG (H&L) conjugated IRDye 800 (Rockland immunochemicals) used at 1:1000 and polyclonal goat anti-mouse IgG (H&L) conjugated Alexa Fluor 680 (Thermo Fisher) used at 1:1000.

Fluorescence *in situ* hybridization. RNAI foci were detected using the protocol described previously (Tran *et al.,* 2015). Salivary glands were dissected and fixed in 4% PFA for 20 minutes. Samples were washed three times in RNase-free PBS containing 0.1% Tween-20 and then permeabilized with 0.5% Triton X-100 and further incubated for 30 minutes in prehybridization buffer (2X Saline Sodium Citrate (SSC), 40% formamide, salmon sperm DNA (1mg/ml) and 0.1% Tween). Samples were incubated for 2 hours at 55°C in prehybridization buffer containing an Alexa488-labelled (G2C4)4 RNA probe (0.2ng/µl). Samples were then washed in pre-warmed wash buffer A (40% formamide, 2X SSC and 0.1% Tween-20) for 30 minutes at 55°C and twice in wash buffer B (2X SSC, 0.1% Tween-20) for 30 minutes at room temperature. After washing with 0.1% Tween-20, samples were blocked and probed as described for IHC for mouse anti-phospho-RNA polymerase II 4H8 (1:500, Abcam) and secondary antibody used was Alexa Fluor 568 (Life Technologies).

Immunohistochemistry. Larval central nervous system, larval and pupal eye discs and adult brains were dissected in chilled PBS Samples were fixed in 2% paraformaldehyde (PFA) for 1 hour and washed in PBS with 0.5% Triton X-100 (PBT). Samples were blocked in 10% normal goat serum (NGS) in PBT for 15 minutes and then incubated with primary antibodies in PBT-NGS overnight at 4°C. Samples were washed in PBT and incubated with secondary antibody in PBT-NGS for 3 hours at room temperature. After washing with PBT and then PBS, samples were stored

in Vectashield with DAPI (Vector laboratories). After mounting, images were taken using the Leica TCS SP5 confocal microscope with Leica Application Suite Advanced Fluorescence (LAS-AF) software (version 2.0.2). Salivary gland preparations were done out as previously described (Zhang *et al.*, 2015) but with fixation in 4% PFA and blocked for 1 hour. Primary antibodies were used at the following concentrations; rabbit anti-GFP 1:1000 (Invitrogen), rabbit polyclonal anti-GP (1:1000, this work), mouse monoclonal anti-GA (1:500, Merck Millipore), anti-GR rat monoclonal (1:300, Merck Millipore), mouse anti-HA clone 6E2 (1:500, Cell Signal), rabbit anti-TBPH (1:2000) (Diaper *et al.*, 2013), rabbit anti-RanGAP (1:300, kind gift from C. Staber); rabbit anti-Importin-α3 (1:300, kind gift from S. Cotterill); rabbit anti-Pendulin (1:300) and mouse anti-dRCC1 (1:10) (both kind gifts from M. Frasch); mouse monoclonal anti-MAB414 (1:500, Abcam), rabbit anti-NUP50 (1:10000, kind gift from J. Großhans), and rabbit anti-REF2P (1:1000, a kind gift from D. Contamine). Secondary antibodies conjugated to Alexa Fluor 488, 568 and 647 (Life Technologies) were used at a final concentration of 1:150.

Image acquisition and analysis. Images were obtained either with Motic BA400 or Leica TCS SP5 confocal microscope with Leica Application Suite Advanced Fluorescence (LAS AF) version 2.0.2 software. For confocal images, channels were scanned sequentially, and confocal z-stacks were processed using FIJI. Figures were arranged using Adobe Illustrator and Adobe Photoshop. For quantification of the TBPH nuclear/cytoplasmic ratio, brains were dissected and washed in primary antibody solution. Single plane images were taken from the medulla of the adult brain always analyzing the same region. Nuclear was defined by DAPI labeling, TBPH immunolabeling was distinguished by nuclear/DAPI and non-nuclear/non-DAPI. 8 brains of each genotype were used for measurements which were carried out using ImageJ.

Eye sectioning. 35 days old males' eyes were incubated in a fixative solution (2% glutaraldehyde in phosphate buffer 0.1M). Same volume of post-fixative solution was added (50% osmium, 50% phosphate buffer 0.2M) for 30 minutes on ice. Samples were then placed in 100% post-fixation solution for 5 hours. Progressive dehydration by ethanol (30%, 50%, 70%, 90%, 100%) was proceeded prior to two propylene oxide washes at room temperature. Eyes were then incubated in mix (1:1) of propylene oxide and epoxy resin overnight. Retinas were embedded the following day in 100% epoxy resin between 4 and 6 hours at room temperature and baked at 80°C overnight. Sections of 1µm sections were performed using a microtome (Leica Ultracut UTC) and stained with 1% toluidine-blue in borax. Images were taken with light microscopy (AxioImager Z1, Zeiss). Quantification was performed by counting the number of photoreceptors on 100 ommatidia on

average per picture using ImageJ. Parraffin eye sections were done as previously described (Zaharieva *et al.,* 2015).

Larval survival. Embryos were collected at 5 hour intervals by replacement of fruit agar plate. L1 larvae were transferred to vials of standard cornmeal media at a density of 50 larvae per vial. Number of flies that underwent complete eclosion were counted. Percentage of flies surviving was calculated based on the original L1 population of 50.

Larval peristalsis. L3 larvae were placed in the centre of fruit agar plates that had been left at room temperature for 30 minutes. L3 larvae were left to acclimatise for 1 minute, after which the number of peristaltic waves that occurred in following minute were counted.

Startle-induced negative geotaxis (SING). Mated female flies were briefly anaesthetized and placed in modified 25mL serological pipettes (Costar) in groups of 15 (n). Flies were left to acclimatise at 25°C for 30 minutes before being tapped to the bottom of the tube and left for 45 seconds with the pipette fixed in an upright position. Following this test run, the same flies were subject to the same stimulus a further 3 times with a 90 second rest between each test. For each test, the number of flies present at the top of the tube (above the 25mL graduation, t), the bottom of the tube (below the 2mL graduation, b) and the middle of the tube (between 2 and 25mL graduations, m) were counted 45 seconds after stimulus delivery. From the 3 repeats for each group of flies, mean values were taken for n, t, b and performance index (PI) scores were calculated for each of the 3 repeats as previously described (White *et al.,* 2010).

Video-assisted motion tracking. Activity tracking was carried out as previously described (White *et al.,* 2010). In addition, activity was defined as movement per frame above a velocity of 2mm/second. Raster plots and activity per minute graphs were generated in MATLAB using custom script. Percentage of time active over the recording period was calculated for each fly. These data were exported to GraphPad Prism 6 for statistical analysis.

Human post mortem tissue analysis. Brain tissue samples were provided from the London Neurodegenerative Diseases Brain Bank (King's College London, UK). Consent for autopsy, neuropathological assessment and research were obtained and all studies were carried out under the ethical approval of the tissue bank (08/MRE09/38+5). Block taking for histological and immunohistochemical studies and neuropathological assessment for neurodegenerative diseases was

performed in accordance with standard criteria. 15 cases were used for the western blot experiments, 5 FTLD-TDP without the *C9ORF72* expansion, 5 FTLD-TDP with confirmed *C9ORF72* expansion and 5 control cases. Controls were defined as subjects with no clinical history and no neuropathological evidence of a neurodegenerative condition.

For western blot analysis, fresh-frozen post-mortem tissue was homogenized in 10 v/w of high salt buffer [100mM 2-(N-morpholino) ethane sulphonic acid (MES) (pH 7.4), 0.5mM MgCl2, 1mM EGTA, 1M NaCl, 50mM imidazole, protease inhibitor cocktail (Roche)]. The homogenate was mixed with 2% sodium dodecyl sulphate polyacrylamide gel electrophoresis loading buffer and boiled for 10 min. Samples were centrifuged for 20 min at 13 000 rpm and 4°C. Equal volumes of samples were loaded on 26-well NuPAGE Novex 10% Bis–Tris pre-cast gels (Invitrogen). Western blots were performed as previously described (Nishimura *et al.*, 2010) using goat anti-KPNA4 (1:750, Abcam) and rabbit anti-GAPDH (1:5000; Cell Signaling Technology). For detection of insoluble protein, the same protocol was used as for fly tissue.

For immunohistochemistry and immunofluorescence of post-mortem human brain samples, the protocols were carried out as previously described (Maekawa *et al.*, 2004). Briefly, 7 μm thick sections from the frontal cortex were cut from formalin-fixed paraffin-embedded tissue blocks and sections were microwaved in citrate buffer to enhance antigen retrieval. For immunofluorescence, primary antibodies were used at the following concentrations; rabbit polyclonal anti-GP (1:1000, this study); mouse monoclonal anti-GA (1:500, Merck Millipore); anti-GR rat monoclonal (1:50, Merck Millipore); rabbit phospho-TDP-43 (1:500, pS409/410-1; 2B Scientific); mouse phospho-TDP-43 (1:500, pS409/410-1, Cosmo Bio Co., LTD); rabbit anti-KPNA4 (1:250, Novus). Secondary antibodies conjugated to Alexa Fluor 488, 568 and 647 (Life Technologies) were used at a final concentration of 1:250. Autofluoresence was quenched using Sudan black treatment and sections were mounted using Vectashield with DAPI. For immunohistochemistry, primary antibody rabbit anti-KPNA4 (1:500, Novus) was applied overnight at 4°C. Following washes, sections were incubated with biotinylated secondary antibody (DAKO), followed by avidin:biotinylated enzyme complex (Vectastain Elite ABC kit, Vector Laboratories, Peterborough, UK). Finally sections were incubated for 5–10 minutes with 0.5 mg/mL 3,3′-diaminobenzidine chromogen (Sigma-Aldrich Company Ltd, Dorset UK) in Tris-buffered saline (pH 7.6) containing 0.05% H_2O_2. Sections were counterstained with Harris' haematoxylin. Immunostaining (morphology, location and intensity) was examined and assessed by a consultant neuropathologist (T.H.) blind to diagnosis.

Statistical analysis. GraphPad Prism 6 was used to perform the statistical analyses indicated within the results section. Comparison of means was performed using either an unpaired t-test, the one-way analysis of variance (ANOVA) or the two-way ANOVA or Bonferroni's multiple

comparisons test. Post-hoc analysis was conducted using Fisher's least significant difference (LSD) test or the Bonferroni-Holm method (Holm, 1979). For categorical data, Fisher's exact test was preferred to Pearson's chi-squared test due to small sample sizes in this dataset.
